# A Membrane-bound eIF2 Alpha Kinase Located in Endosomes Is Regulated by Heme and Controls Differentiation and ROS Levels in *Trypanosoma cruzi*

**DOI:** 10.1371/journal.ppat.1004618

**Published:** 2015-02-06

**Authors:** Leonardo da Silva Augusto, Nilmar Silvio Moretti, Thiago Cesar Prata Ramos, Teresa Cristina Leandro de Jesus, Min Zhang, Beatriz A. Castilho, Sergio Schenkman

**Affiliations:** 1 Departamento de Microbiologia, Imunologia e Parasitologia, Escola Paulista de Medicina, Universidade Federal de São Paulo, São Paulo, São Paulo, Brazil; 2 Department of Pathology, New York University School of Medicine, New York, New York, United States of America; London School of Hygiene & Tropical Medicine, UNITED KINGDOM

## Abstract

Translation initiation has been described as a key step for the control of growth and differentiation of several protozoan parasites in response to environmental changes. This occurs by the activation of protein kinases that phosphorylate the alpha subunit of the translation initiation factor 2 (eIF2α), which decreases translation, and in higher eukaryotes favors the expression of stress remedial response genes. However, very little is known about the signals that activate eIF2α kinases in protozoan parasites. Here, we characterized an eIF2α kinase of *Trypanosoma cruzi* (TcK2), the agent of Chagas’ disease, as a transmembrane protein located in organelles that accumulate nutrients in proliferating parasite forms. We found that heme binds specifically to the catalytic domain of the kinase, inhibiting its activity. In the absence of heme, TcK2 is activated, arresting cell growth and inducing differentiation of proliferative into infective and non-proliferative forms. Parasites lacking TcK2 lose this differentiation capacity and heme is not stored in reserve organelles, remaining in the cytosol. TcK2 null cells display growth deficiencies, accumulating hydrogen peroxide that drives the generation of reactive oxygen species. The augmented level of hydrogen peroxide occurs as a consequence of increased superoxide dismutase activity and decreased peroxide activity. These phenotypes could be reverted by the re-expression of the wild type but not of a TcK2 dead mutant. These findings indicate that heme is a key factor for the growth control and differentiation through regulation of an unusual type of eIF2α kinase in *T. cruzi*.

## Author Summary

*Trypanosoma cruzi* proliferates as epimastigotes in the midgut of the insect vector filled with blood meal. There, it accumulates nutrients in specific endosomal organelles. The parasite moves towards the hindgut and when the blood is completely digested, these organelles are consumed. At this moment, the insect is ready for a new feeding cycle that promotes the release of infective metacyclic-trypomastigote forms. We have previously found that such differentiation involves protein synthesis arrest through the phosphorylation of the eukaryotic translation initiation factor 2α (eIF2α). Now, we show that one of the kinases (TCK2) that phosphorylate eIF2α is localized in these endosomes. TcK2 binds and is specifically inhibited by heme derived from blood hemoglobin. We also found that heme inhibits differentiation, suggesting that it is an important signal for differentiation. By generating knockouts of TcK2, we observed an increased accumulation of heme in the cytosol, which induced cellular damage by affecting the reactive oxygen metabolism in the parasite. We conclude that this eIF2α kinase senses cytosolic heme obtained from the blood meal, promoting its storage in the cytosolic organelles. When heme levels are decreased in the cytosol, TcK2 activation can then arrest protein synthesis that is followed by the induction of the differentiation of proliferative epimastigote forms to infective metacyclic-trypomastigotes.

## Introduction

The phosphorylation of the alpha subunit of the trimeric eukaryotic initiation factor 2 (eIF2) complex is a key event in the cellular stress response [1]. This phosphorylation inhibits the conversion of GDP to GTP in eIF2 by the eIF2B, a guanosine exchange factor, thus decreasing the levels of the ternary complex eIF2-GTP-Met-tRNA^i^ available for new rounds of translation initiation. At the same time that global translation is inhibited, there is preferential translation of specific messages that promote the remediation from stresses in several eukaryotes [2]. The phosphorylation of eIF2α is catalyzed by serine-threonine kinases specifically activated by the different stress conditions [3]. Mammals have four kinases that phosphorylate eIF2α: a) The general control nonderepressible 2 (GCN2) kinase (KEGG K16196, also known as eIF2AK4), which is activated by amino acid deprivation through binding to uncharged tRNAs [4]. b) The RNA-activated protein kinase (PKR) (KEGG 16195, also known as eIF2AK2), which is activated by double strand RNA in cells infected with viruses [5, 6, 7]. c) The PKR-like endoplasmic reticulum kinase (PERK) (KEGG K08860, also known as eIF2AK3), which is activated by unfolded proteins in the endoplasmic reticulum [8]. d) The heme-regulated eIF2α kinase (HRI) (KEGG K16194, also known as eIF2AK1), which is activated by heme deficiency [9]. Different stress conditions result in conformational changes within the catalytic domain of these kinases leading to autophosphorylation and activation, allowing for the binding and subsequent phosphorylation of the eIF2α substrate.

Similar kinases of eIF2α are present in other organisms and are involved in stress sensing and remediation [10]. While GCN2, or a GCN2-like kinase, is present in virtually all eukaryotic organisms, other eIF2α kinases are more scattered through different organisms. For example, HRI is present in some fungi including *Schizosaccharomyces pombe*. PERK orthologues have been identified in worms, insects and vertebrates. Specific phosphorylation of eIF2α by unique kinases is also directly involved in responses to environmental changes throughout the lifecycle of protozoan parasites (reviewed in [11, 12]). For example, the eIF2α kinase eIK2 controls the latency of *Plasmodium* sporozoites in insect salivary glands through eIF2α phosphorylation [13]. The eIK1 is homologous to GCN2, as it is activated by amino acid starvation, regulating the erythrocyte cycle of *Plasmodium* [14]. The PK4 has a topology similar to PERK and controls the growth of schizonts and gametocytes of *Plasmodium* [15]. In *Toxoplasma gondii* eIF2α phosphorylation takes place during the differentiation to bradyzoites, which remain latent in the vertebrate host, or during parasite passage through the extracellular medium, when the parasite is subjected to lower amino acid levels [16, 17, 18].

The phosphorylation of eIF2α is also involved in the control of trypanosome differentiation in response to variations in nutrients and temperature. In these organisms, there are three putative eIF2α kinases: a) K1, which is similar to GCN2, b) K2 with a topology similar to PERK, and c) K3, with little similarity to the known eIF2 kinases [19]. The physiological significance of these kinases is mostly unknown. In *Leishmania*, the K2 ortholog (LinPERK) is localized in the endoplasmic reticulum and regulates the differentiation of promastigotes into amastigotes by unknown stimulus [20, 21]. Interestingly, in *Trypanosoma brucei* the homologous kinase (TbeIF2αK2) is localized in the flagellar pocket membrane, site of intense membrane internalization, and early endocytic vesicles [19]. TbeIF2αK3 has been recently shown to migrate from the endoplasmic reticulum to the nucleus upon persistent stress, abrogating transcription events [22]. In *Trypanosoma cruzi*, we have demonstrated that eIF2α phosphorylation is required for the differentiation of the non-infective epimastigotes into infective metacyclic trypomastigotes (metacyclogenesis) stimulated by starvation [23], mimicking what happens in the insect hindgut [24, 25]. Metacyclogenesis takes about 72 h to be accomplished when the parasites finally cease translation [23]. As nutrients stored in endosome-like organelles, also called reservosomes, are consumed during metacyclogenesis [26], we presumed that TcK2, the kinase homologous to the *T. brucei* flagellar pocket eIF2 kinase (TbeIF2αK2) might be involved in *T. cruzi* differentiation. Herein, we studied the expression and the localization of TcK2 kinase in different life stages of the parasite and generated knockout parasites of this kinase. We provide evidence that in *T. cruzi*, TcK2 is located in the endosomal compartment and it is regulated by heme, controlling the parasite’s growth and differentiation.

## Results

### TcK2 is a membrane bound eIF2α kinase that undergoes autophosphorylation and phosphorylates the parasite eIF2α

**Figure 1:**
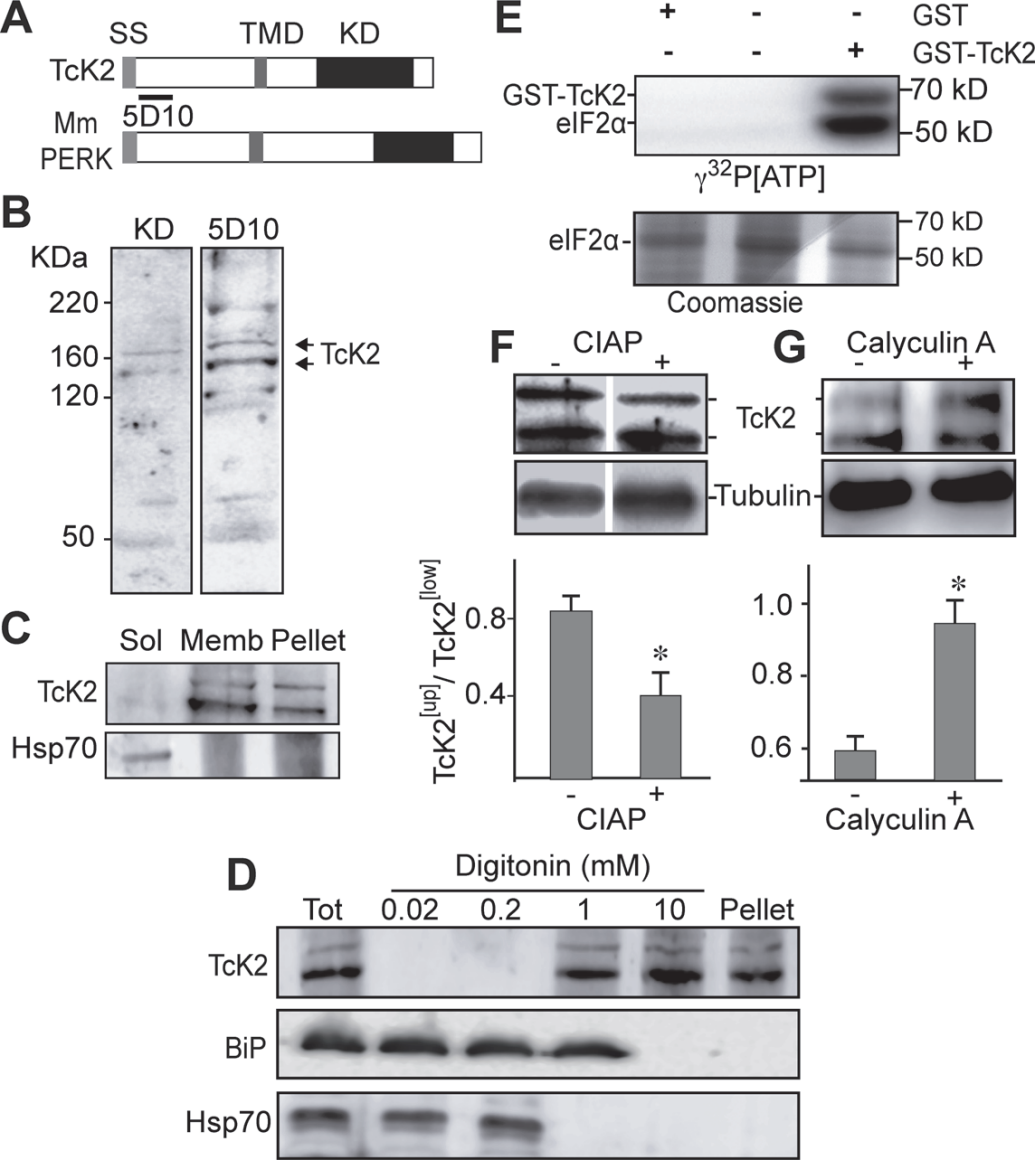
*T. cruzi* K2 is a membrane bound protein kinase that undergoes autophosphorylation and phosphorylates the parasite eIF2α. (A) Comparative topology of *T. cruzi* (TcK2) and *Mus musculus* (Mm) PERK indicating the signal sequence (SS), the transmembrane (TMD) and the kinase (KD) domains. (B) Western blot of total extracts of *T. cruzi* epimastigotes incubated with an antiserum from a rabbit immunized with a recombinant protein corresponding to *T. brucei* K2 KD or with a mAb 5D10 prepared against a recombinant K2 fragment located as indicated in panel (A). (C) Western blot of the 100,000 g supernatant (Sol) (1 h centrifugation) from parasites extracts; of the supernatant from the previous pellet after extraction with 1% Triton X100 in the same buffer extraction (Memb) and of the insoluble fraction after detergent extraction (Pellet). *T. cruzi* epimastigotes were lysed in 0.1 M NaCl, 50 mM Tris-HCl (pH 7.4), 2 mM MgCl_2_, 10 mM ethylene-glycol tetra-acetic acid, containing the cOmplete cocktail by three freezing and thawing cycles. The blots were probed with anti-TcK2-KD and anti-Hsp70 antibodies. (D) Western blot of lysates of epimastigotes (Tot), or the supernatants of epimastigotes lysed in the presence of indicted amounts of digitonin as described [21] and probed with and anti-TcK2 and anti-BiP and anti-HsP70 antibodies. (E) SDS-PAGE of 6XHis-tagged TceIF2α incubated in the presence of indicated proteins for 15 min with [^32^P]-γ-ATP. The upper panel shows the autoradiogram and the bottom panel the same gel stained with Coomassie Blue R250. (F) and (G) are blots of, respectively, total extracts of *T. cruzi* epimastigotes treated with CIAP or of cells pre-incubated for 2 h without (−) or with (+) 10 nM of calyculin A. Anti-β-tubulin was used as a loading control. The histograms below the gel represent the relative amounts of the upper phosphorylated band of TcK2 (TcK2^[Up]^) and the fast migrating band of TcK2 (TcK2^[Low]^). The values are mean and standard deviations of triplicates. Asterisks indicate statistically significant differences (p < 0.05) based on t-Student test.

The predicted topology of *T. cruzi* K2 was found similar to mammalian PERK. TcK2 contains a kinase domain at its C-terminal, a transmembrane domain in the middle, and a signal peptide at its N-terminal (Fig. 1A). Polyclonal antibodies generated against the kinase domain of TbeIF2αK2 [19] and a monoclonal antibody (mAb) 5D10 obtained from mice immunized with the TcK2 N-terminal region recognized specifically two bands (140–160 kDa) in Western blots of total parasite lysates (Fig. 1B). The other bands seen in the Western blot were either degradation products, or non-specific antibody labeling, as they were not seen in all experiments. The protein is associated with parasite membranes as it could be solubilized only in the presence of detergents, in contrast to the soluble cytosolic heat shock protein (Hsp) 70 (Fig. 1C). Moreover, TcK2 was only solubilized in high concentrations of digitonin, differently from BiP, present in the endoplasmic reticulum lumen or the Hsp70, located in the cell cytosol (Fig. 1D).

The kinase domain of TcK2 fused with the glutathione S-transferase (GST) was expressed in *E. coli*. The recombinant GST-TcK2, but not GST, obtained from *E. coli* was able to phosphorylate the purified His6x-tagged-TceIF2α *in vitro*, as shown in Fig. 1E. In addition, it became phosphorylated, as indicated by the presence of the second labeled band in the autoradiogram with the expected size of GST-TcK2 (68 kDa). Phosphorylation of TcK2 was also suggested to occur *in vivo*, as treatment of cell lysates with calf intestine alkaline phosphatase (CIAP) decreased the relative intensity of the slow migrating band (Fig. 1F). In addition, pre-treatment of live parasites with calyculin A, a general phosphatase inhibitor, increased the levels of the slow migrating band. These observations indicated that this slow migrating band might correspond to the autophosphorylated and active form of the TcK2 kinase.

### *T. cruzi* K2 is localized in endosomes and the upper phosphorylated band predominates in non-proliferative stages

**Figure 2:**
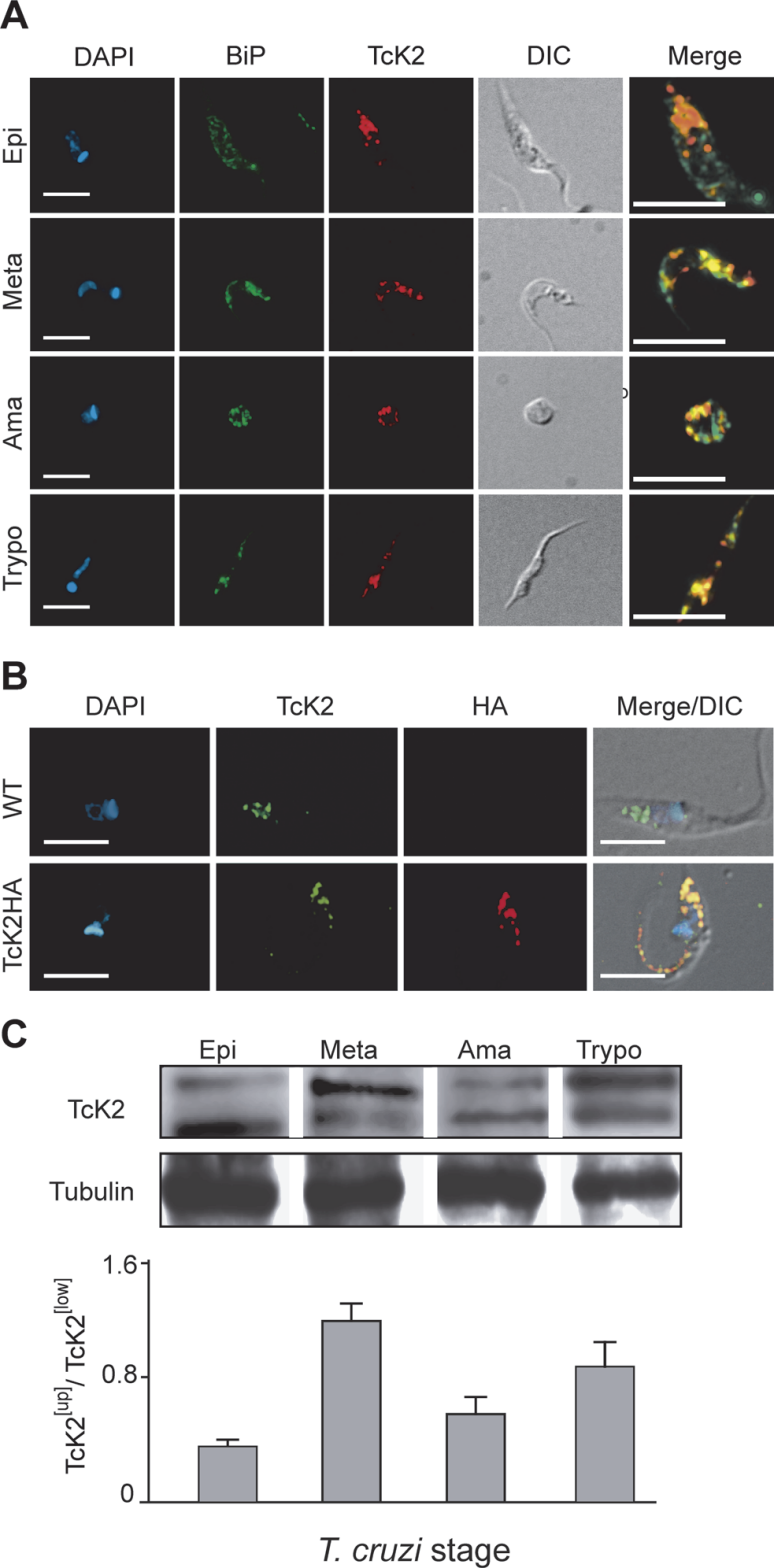
TcK2 is located in the endosomal compartment and is phosphorylated in non-proliferating parasite stages of *T. cruzi*. (A) Wild type epimastigotes (Epi), metacyclics (Meta), intracellular amastigotes (Ama) and cell-released trypomastigotes (Trypo) were fixed, permeabilized, and incubated with anti-BiP antibodies (Bip, green) and mAb 5D10 (TcK2, red). The images show individual immunofluorescence and DAPI labeling, DIC images and the merged immunofluorescence labeling from an enlarged field (Bars = 5 μm). (B) Wild type (WT) or HA-tagged K2 (TcK2-HA) epimastigotes were adsorbed to glass slides, fixed with paraformaldehyde and permeabilized with 0.1% Triton X100. Adhered cells were then stained with mAb 5D10 (TcK2, green) or anti-HA (HA, red), and DAPI (blue). The images show individual labeling and the fluorescence signals merged with differential interference contrast (DIC). (C) Western blots of total extracts of parasites of the indicated stages revealed by anti-TcK2 (anti-KD) antibody. Anti-β-tubulin was used as a loading control. The graph shows the mean and standard deviation of the TcK2^[Up]^/TcK2^[Low]^ ratio (n = 3).

Immunofluorescence showed that TcK2 is located in cellular organelles in the epimastigote form of the parasite (Fig. 2A). This form corresponds to the stage that proliferates in the gut of the insect vector. The same result was obtained with a C-terminal HA-tagged TcK2 expressed in epimastigotes (Fig. 2B). TcK2 was found expressed in both replicative and infective forms (Fig. 2A and 2C). In epimastigotes and amastigotes, respectively the forms that proliferate in the insect gut and in the cytosol of mammalian cells, the enzyme showed poor colocalization with BiP, an ER-resident protein (Fig. 2A). Increased, but incomplete colocalization with BiP was observed in trypomastigote stages, which are the infective and non-proliferative forms. Interestingly, in proliferating epimastigotes and amastigotes, the lower band, which may correspond to the non-phosphorylated form of TcK2, predominated while in the infective forms (metacyclic-trypomastigotes and cell-derived trypomastigotes) the upper phosphorylated band was predominant (Fig. 2C), suggesting that the kinase could be more active in the infective forms.

### Hemin starvation causes an increase in the slow migrating TcK2 form

**Figure 3:**
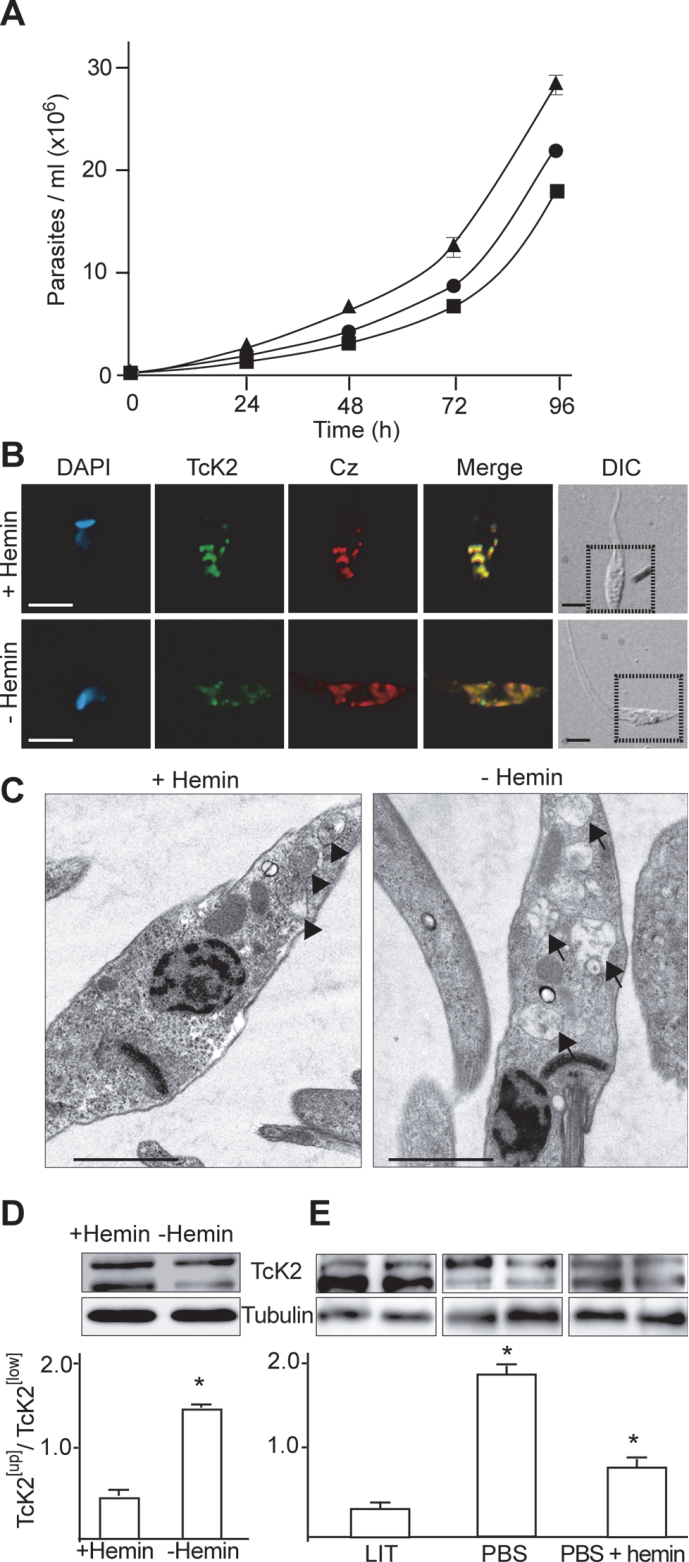
Heme promotes parasite multiplication with accumulation of electron dense endosomes and changes in TcK2 gel migration. (A) Epimastigotes were cultivated in LIT medium prepared without adding hemin (black circle), or with 10 μg (black square) or 30 μg (black triangle) of hemin per ml and counted along the indicated times. Values are mean and standard deviation (n = 3). (B) Epimastigotes were maintained for 96 h in the absence of added hemin (−Hemin), or in the presence of 30 μg/ml of hemin, and processed for immunofluorescence with mAb 5D10, or with anti-cruzipain (Cz). The images also show the DAPI staining; the merged immunofluorescences of the two antibodies, and the corresponding field marked with dotted squares in the respective DIC images (Bars = 5 μm). (C) Transmission electron micrographs of parasites maintained in LIT prepared with 30 μg/ml of hemin (+Hemin) or without hemin supplementation (−Hemin) (Bars = 2 μm). Arrowheads indicate the reservosomes filled with electron dense material and arrows indicate the same structures with less electron dense material. Western blots using anti-TcK2-KD and anti-β-tubulin antibodies of samples prepared from parasites kept in the medium without (−) or with (+) hemin supplementation (30 μg/ml) (D), or from parasites kept 6 h in LIT medium containing hemin (10 μg/ml), PBS, or PBS with 30 μg/ml of hemin (E). The gel shows biological duplicates and below are the means and standard deviation of the TcK2 ^[Up]^/TcK2^[Low]^ (n = 3). The differences are statistically significant (p < 0.05) based on the t-Student test.

Epimastigotes proliferate in the insect gut, a compartment full of blood meal. The parasite stores different nutrients, including heme [27] and lipids, such as cholesterol [28], in organelles derived from endosomes called reservosomes [29]. As nutrients become scarce, differentiation of epimastigotes into metacyclic-trypomastigotes is triggered and concomitantly these endosomes are consumed [26]. As heme is largely present in the blood meal and is required for epimastigote growth [30], we tested the effect of adding heme in the form of hemin into the culture medium and checked parasite growth, TcK2 localization and the enzyme migration by gel electrophoresis. As shown in Fig. 3A, parasites maintained in heme-rich medium (LIT with hemin) propagated faster than parasites cultivated in medium without heme supplementation, as shown previously [31]. In the heme-rich medium, TcK2 was detected mainly in endosomes, co-localizing with cruzipain (Fig. 3B) a protease present in these organelles [32]. Both proteins were found more diffuse in the parasites maintained in media with reduced amounts of hemin (Fig. 3B). This is compatible with the different solubilization of TcK2 by digitonin in trypomastigote forms when compared to epimastigotes (S1 Fig. and Fig. 1D), or when cells were maintained in reduced amounts of hemin (Fig. 2A), suggesting a different localization of TcK2 in the various parasite stages.

In parasites cultivated in medium supplemented with hemin, electron microscopy showed more organelles appearing as electron-dense vesicles, while in hemin poor medium electron-lucent structures predominate (Fig. 3C). These results are in agreement with the proposal that heme causes the elevated electron density of these *T. cruzi* structures, located in the posterior end of the cell, possibly reservosomes, based on incorporation of tracers added externally to parasites [32].

More importantly, in the absence of hemin, the upper band of TcK2 predominated in total parasite lysates (Fig. 3D). Similarly, when parasites were transferred to saline buffer (PBS), the upper band of TcK2 prevailed and this was inhibited by addition of hemin (Fig. 3E). These results indicate that heme accumulation in the endosomes could be related to TcK2 inactivation.

### Hemin binds to and specifically inhibits TcK2

**Figure 4:**
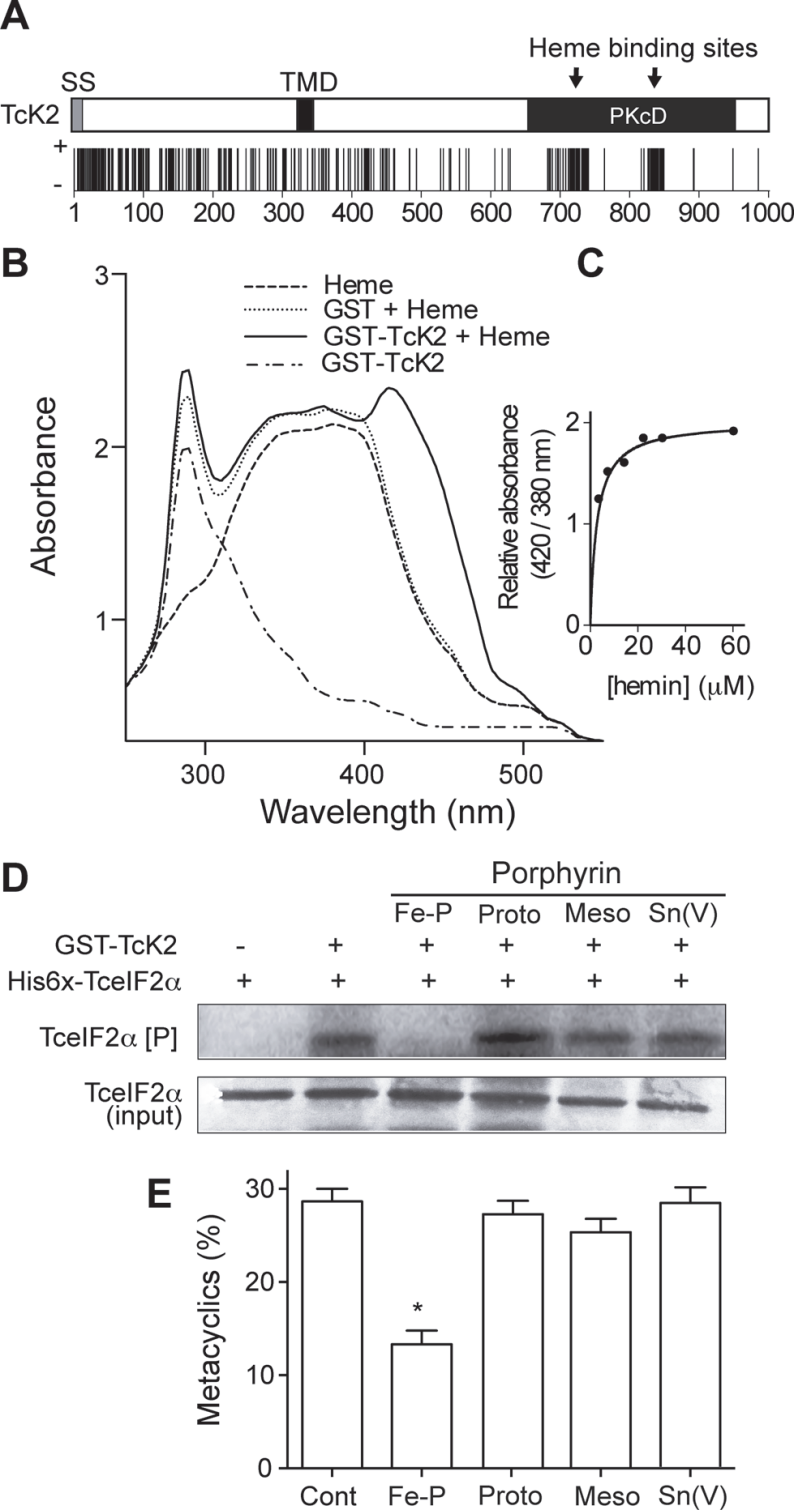
Heme binds specifically to the catalytic domain of TcK2 and inhibits TceIF2α phosphorylation and metacyclogenesis. (A) Prediction of heme binding sites along the TcK2 sequence according to the Hemebind software [33]. The vertical dark bars are positive hits for each amino acid residue. (B) Absorption spectra of 10 μM hemin (dashed line), GST + 10 μM hemin (dotted line) and GST-TcK2 (dotted/dashed line), and GST-TcK2 incubated with 10 μM hemin (continuous line). (C) The figure shows the relative absorbance at the indicated concentrations of hemin incubated with GST-TcK2 at 25°C. (D) SDS-PAGE of His6x-TceIF2α incubated without (−) or with (+) GST-TcK2 for 15 min with [^32^P]-γ-ATP in the absence or presence of 10 μM of the indicated porphyrins. The upper gel shows the autoradiogram and at the bottom is the same gel stained with Coomassie Blue R250. Panel (E) shows the percentages of differentiated cells (metacyclics-trypomastigotes) after 96 h in the presence of the indicated porphyrins counted by direct observation of 250 cells per replicate in the fluorescence microscope after DAPI staining. The numbers are mean and standard deviation (n = 3). The asterisk indicates a significant difference (p < 0.05) based on the t-Student test.

Two putative heme binding sites in the catalytic domain of TcK2 could be predicted based on sequence analysis [33], as shown in Fig. 4A. These sites are represented in more detail as horizontal bars in the alignment of the kinase domain of HRI, PERK and the two kinases of *T. cruzi* (S2 Fig.). Indeed, a GST-TcK2 recombinant protein displayed a typical Soret spectral band, with a peak from 410 to 420 nm in the presence of heme, not seen in the GST-TcK2 alone, as detected by spectroscopy analysis (Fig. 4B). This spectral band is formed in proteins that bind to heme. Quantitative assays using different amounts of hemin revealed that the increase in absorbance at 410 nm was saturable, suggesting specific binding and allowing us to estimate an apparent heme binding constant (Kd^app^) of 2.8 ± 0.6 μM (Fig. 4C).

To test the effect of heme in the regulation of TcK2 activity, we assessed the *in vitro* phosphorylation of TceIF2α by GST-TcK2 in the presence of heme and several analogs. Heme (Fe-Protoporphyrin IX) was able to inhibit the activity of GST-TcK2, while protoporphyrin IX, mesoporphyrin IX, and Sn protoporphyrin had no effect (Fig. 4D). Importantly, this inhibitory effect was specific, as the PK4 kinase from *P. falciparum* did not bind to heme and heme did not inhibit eIF2α phosphorylation by PK4 (S3B Fig.) [15].

When we analyzed the effect of heme and its analogs, we also found that only heme inhibited the differentiation of epimastigotes into metacyclic trypomastigotes (Fig. 4E). As previously reported, the differentiation depends on eIF2α phosphorylation and protein synthesis attenuation [23]. Taken together, these data suggest that heme binding inactivates TcK2, thus inhibiting *T. cruzi* differentiation.

### TcK2 null cells show decreased differentiation and proliferation rates

**Figure 5:**
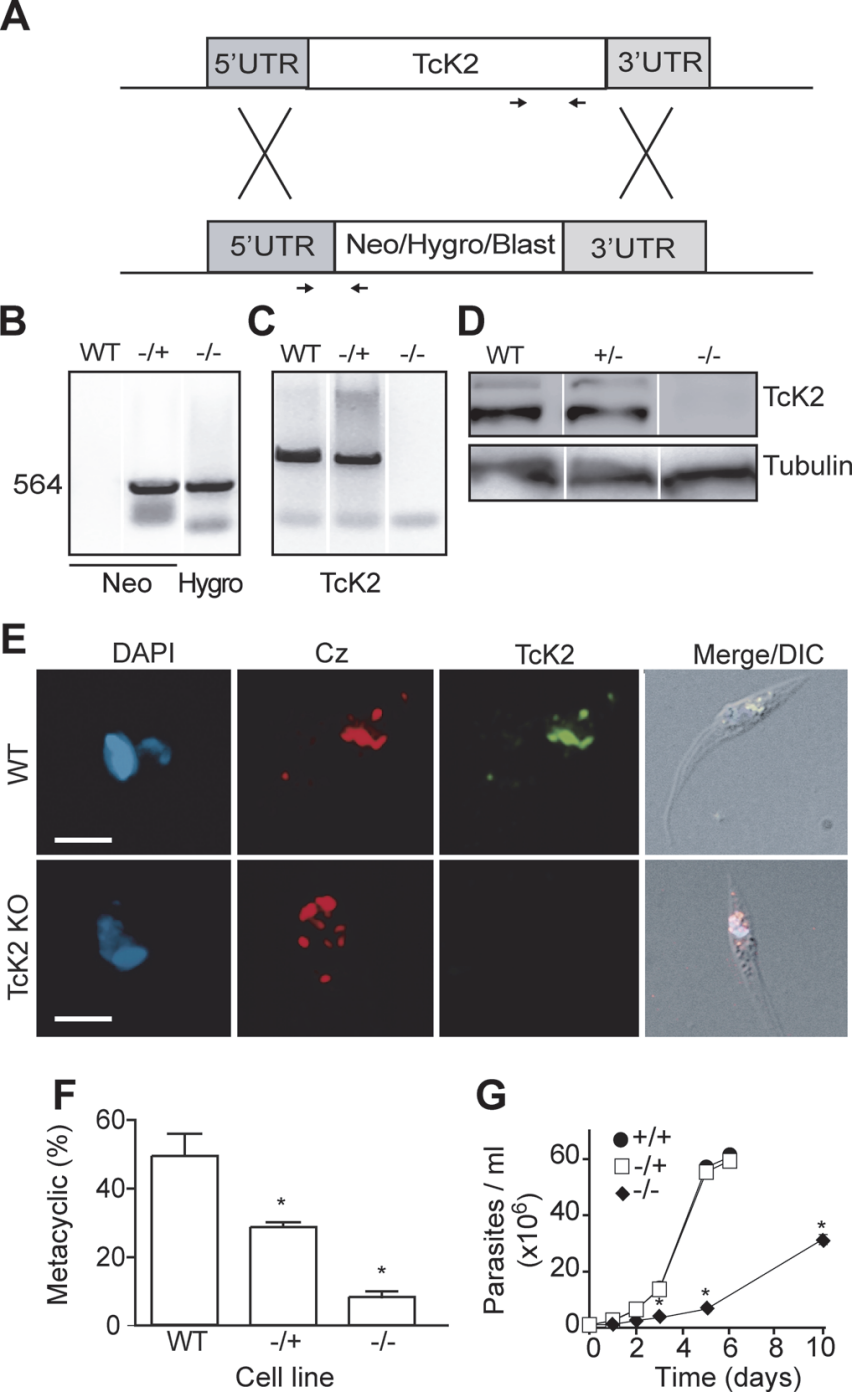
TcK2-KO cells are impaired in cellular differentiation and display reduced growth. (A) Schematic diagram illustrating the homologous recombination strategy to generate knockouts of TcK2. (B) PCR products of specific resistance gene using as a template the DNA extracted from wild type (WT), partial KO (+/−) and the TcK2 null (−/−) parasites. (C) Same as in B, but using oligonucleotides to amplify part of the open reading frame of TcK2. The localization of oligonucleotides used in (B) and (C) is indicated in panel (A). (D) Western blot of extracts obtained from wild type, partial KO (+/−) and TcK2 null (−/−) parasites, using anti-TcK2-KD (top panel) or anti-β-tubulin (bottom panel) antibodies. (E) Immunofluorescence of the wild type and TcK2 null epimastigotes using mAb 5D10 (green) and anti-cruzipain (red) (Bars = 2 μm). (F) Percentages of differentiated (metacyclics-trypomastigotes) obtained from wild type, partial KO (+/−) and TcK2 null (−/−) cells. (G) Growth curve of wild type (black circle), partial KO (white square), and TcK2 null parasites (black losangle). In F and G numbers are mean and standard deviation (n = 3) and asterisks indicate a significant difference (p < 0.05) based on the t-Student test.

To better understand the TcK2 function in the parasite, we generated TcK2-null cell lines by a two-step gene replacement through homologous recombination and antibiotic selection as illustrated in Fig. 5A. The integration of resistance genes (Neo and Hygro, or Blast) in the TcK2 locus of the two homologous chromosomes was confirmed by PCR using specific primers (Fig. 5B). The TcK2 gene was not amplified from the double KOs (−/−) (Fig. 5C) and the cells did not express the kinase as shown by Western blot (Fig. 5D) and immunofluorescence assays (Fig. 5E). TcK2 null cells still expressed cruzipain in organelles (Fig. 5E), showing that the kinase is not necessary for cruzipain incorporation into endosomes. As expected, epimastigotes with deletion of one TcK2 allele (−/+) and the TcK2 null cell line (−/−) showed a progressive inhibition in differentiation to metacyclic-trypomastigotes (Fig. 5F), compatible with the heme starvation followed by eIF2α phosphorylation being a factor inducing differentiation. Strickling, in hemin-containing medium, only the TcK2 null cells showed a reduced growth rate (Fig. 5G). The same phenotypes were obtained in four independent clones, two of them using blasticidin instead of hygromycin resistance genes (see below).

### Ascorbate restores the growth rate and cell viability but not the full differentiation capacity

**Figure 6:**
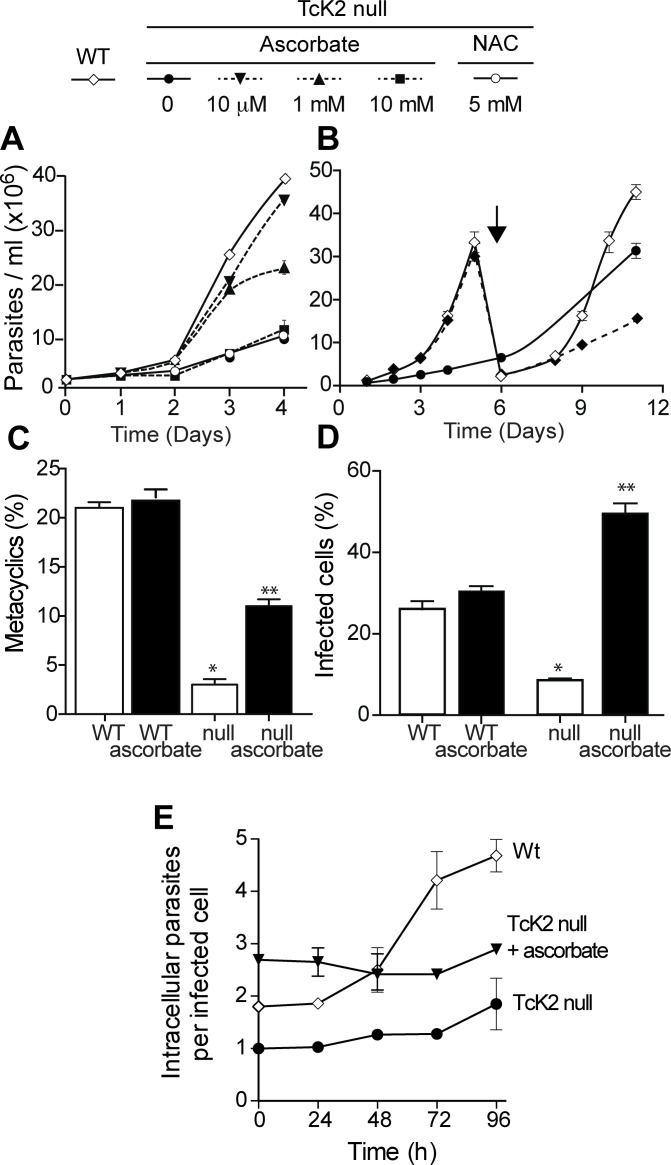
Ascorbate restores TcK2 null parasite growth, differentiation and infectivity, but not intracellular proliferation. (A) Growth of wild type (WT, white losangle) and TcK2 null cells in the absence (black circle) or in the presence of 10 μM (black inverted triangle), 1 mM (black triangle) or 10 mM (black square) ascorbate, or 5 mM N-acetyl-cysteine (white circle). (B) Growth of WT (white losangel) and TcK2 null parasites in the absence of ascorbate (black circle), or after removal of 10 μM ascorbate on day 0 (white losangle). On day 6, the cells were diluted 10 fold (see arrow). (C) and (D) show, respectively, the percentage of cellular differentiation into metacyclics-trypomastigotes after 96 h and the invasion of mammalian cells by the same number of wild type (WT) and the TcK2 null parasites in the absence (empty bars) or presence of 10 μM ascorbate (filled bars). (E) Intracellular proliferation of amastigotes after infection by the wild type (WT, white losangle), TcK2 null cells previously differentiated in the absence (black circle) or in the presence of 10 μM ascorbate (black inverted triangle). In all cases, the values are mean and standard deviation of triplicates. Asterisks indicate statistically significant differences between the WT and null lines in each case based on t-Student test (* p < 0.01, ** p < 0.3).

The slow growth phenotype of the TcK2 null cells could be due to oxidative damage generated by the hemin in parasites lacking this kinase. Therefore, we examined the effect of antioxidant agents in parasite growth. As shown in Fig. 6A, ascorbate restored normal growth when used at concentration of 10 μM; with 1 mM of ascorbate, growth was initially restored but the proliferation rate diminished after a few days. At the concentration of 10 mM, ascorbate was not effective. The effect of ascorbate was not because it restored the levels of sulfhydryl groups, as N-acetyl-cysteine, a sulfhydryl reducing agent, did not restore growth. Moreover, the effect of ascorbate disappeared after three days when cells were washed and maintained in medium without ascorbate (Fig. 6B), suggesting that a factor that prevents growth progressively accumulates in the medium of TcK2-KO cells.

On the other hand, 10 μM ascorbate only partially reverted the poor differentiation capacity of TcK2 null epimastigotes to metacyclic-trypomastigotes (Fig. 6C). This partial reversion of differentiation is suggestive that TcK2 affects differently the growth and the metacyclogenesis. The generated TcK2 null metacyclics also showed a reduced capacity to invade mammalian cells (Fig. 6D). Moreover, after infection, the TcK2 null parasites were able to differentiate into amastigotes but did not grow (Fig. 6E), or produce trypomastigotes, even after long incubations. Also, when metacyclics-trypomastigotes from TcK2 null cells were produced in the presence of ascorbate, the invasion was largely increased (Fig. 6D), without intracellular multiplication (Fig. 6E).

### TcK2 null cells display more electron lucent structures

**Figure 7:**
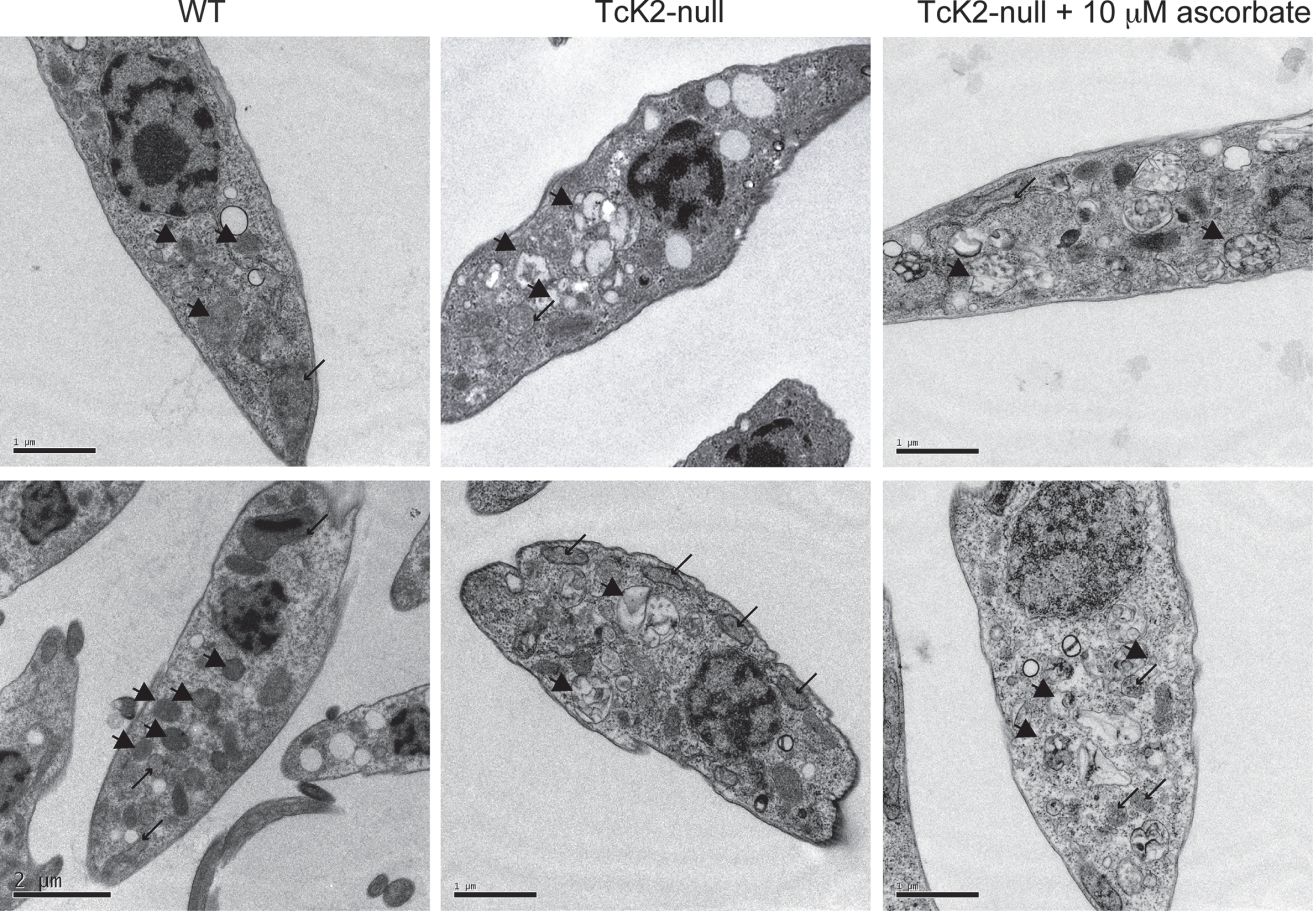
TcK2 null parasites lose electron dense organelles and their contents are not restored by ascorbate. Transmission electron micrographs of wild type (WT), TcK2 null and TcK2 null parasites maintained in 10 μM ascorbate (Bars = 2 μm). Arrowheads indicate the endosomal organelles and thin arrows sections of the mitochondrion in all cases.

The TcK2 null cells still showed organelles that resemble reservosomes, as revealed by the cruzipain signal in the immunofluorescence analysis (Fig. 5E). Indeed, TcK2 null parasites examined by transmission electron microscopy showed cytoplasmic organelles that were more electron-lucent than in wild type parasites (Fig. 7). The empty structures were also found in parasites cultivated in medium without hemin supplementation (Fig. 3C), suggesting that heme does not accumulate in these organelles in cells lacking TcK2. These structures were clearly distinguishable from the mitochondrion, which was identified by the double membranes. Remarkably, in TcK2 null parasites treated with 10 μM ascorbate, which restored epimastigotes growth, these organelles remained electron-lucent, probably not accumulating heme. This observation suggests that the reduced proliferation in the absence of ascorbate was not because of the lack of heme in the endosomal compartment.

### Heme levels and superoxide dismutase activity increase and peroxidase decreases in TcK2 null parasites

**Figure 8:**
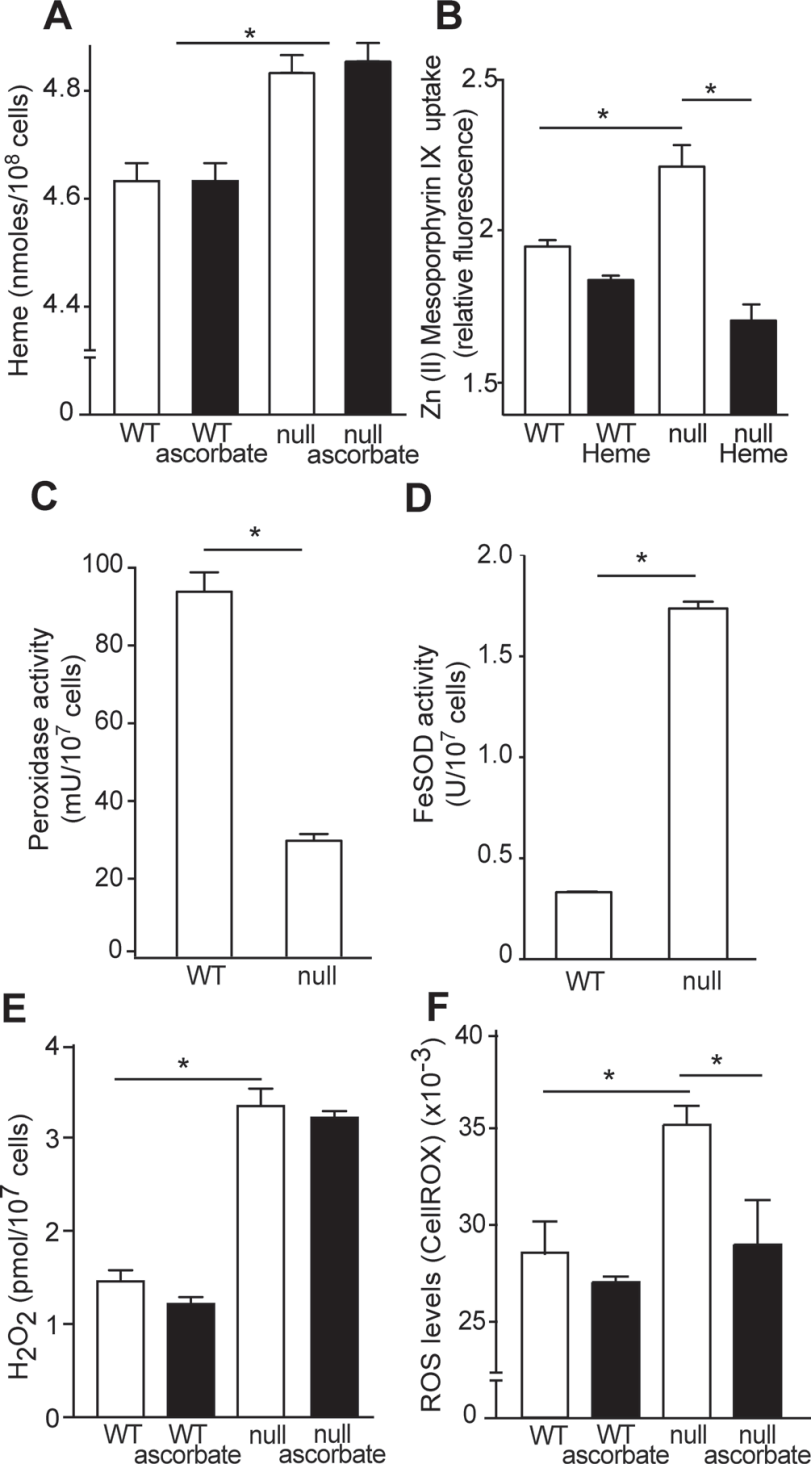
TcK2 null parasites incorporate more heme than WT cells, resulting in increased H_2_O_2_ and ROS levels. (A) Intracellular heme levels in wild type (WT) and TcK2 null parasites growing in the absence (empty bars) or in the presence (filled bars) of 10 μM ascorbate. (B) Zn (II) mesoporphyrin IX incorporation measured in WT and TcK2 null cells in the absence (empty bars) or in the presence (filled bars) of 10 μM heme. (C) and (D) respectively indicate the total peroxidase and FeSOD activities in WT and TcK2 null parasites. (E) and (F) respectively show the H_2_O_2_ and ROS levels in WT and TcK2 null cells cultivated in the absence (empty bars) or presence (filled bars) of 10 μM ascorbate. In all cases, the values are mean and standard deviation of triplicates. Asterisks indicate statistically significant differences based on t-Student test (p < 0.01).

Next, we quantified the total amount of heme in the parasites after gentle lysis, expecting to measure mainly molecules present in the cytosol. The TcK2 null parasites had larger amounts of heme than wild type parasites, and this increase was not reversed by ascorbate, as shown in Fig. 8A. We also observed a larger uptake of the fluorescent heme analogue [Zn(II) Mesoporphyrin IX] by the TcK2 null cells when compared to the wild type cells. This uptake was strongly inhibited by heme in TcK2 null parasites as compared to wild type parasites, ruling out a non-specific binding to the cell (Fig. 8B). These results suggest that TcK2 could act in sensing and/or regulating the amount of heme that is present in the cells. Together with the electron-microscopy results, such data indicate that in parasites lacking TcK2, heme taken up by the cell is not directed to the lumen of endosomes, remaining in the cytosol.

The absence of TcK2 also caused a decrease in the total peroxidase activity (Fig. 8C), and an increase in the Fe-superoxide dismutase (SOD) activity when compared to wild type parasites (Fig. 8D). Consequently, the total levels of hydrogen peroxide were largely increased (Fig. 8E), independently of the presence of ascorbate. However, there was an increase in the amount of reactive oxygen species (ROS), as measured using CellRox (Fig. 8F), which was reduced by ascorbate addition. An increase from 600 fluorescence units in the case of control and 1000 fluorescence units corresponding to ROS levels was also detected using 5CM-H2DCFDA. However, this reagent provided a much higher background, due to the presence of medium. Anyway, the reduced growth of the TcK2 null cells correlated with the increased levels of ROS, probably produced by the augmented levels of H_2_O_2_, which might react with free iron or copper to generate hydroxyl radicals, causing parasite damage.

### Only the active kinase restores the wild type phenotypes in TcK2 null parasites

**Figure 9:**
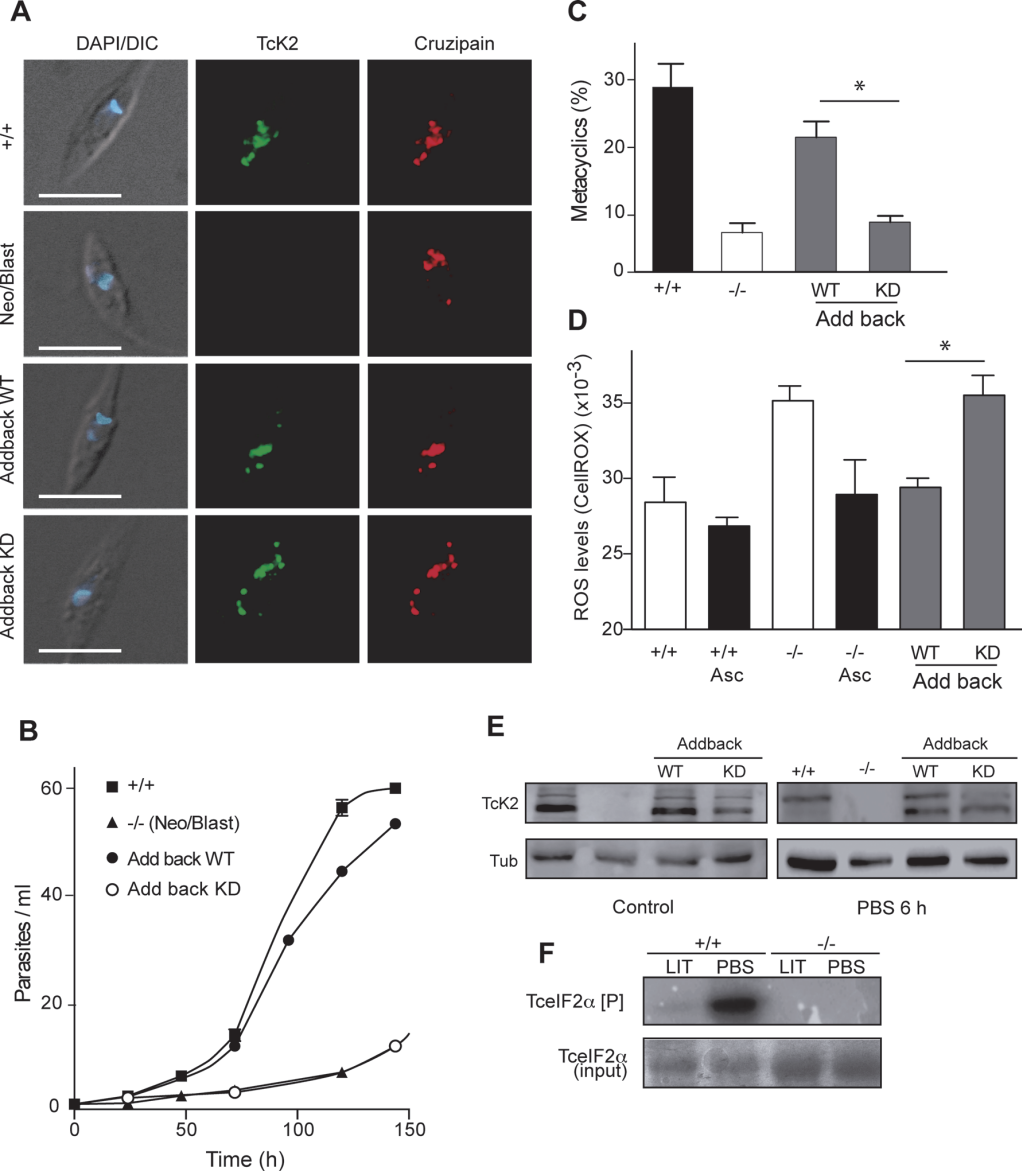
Wild type, but not the kinase dead TcK2 restores the knockout phenotypes. (A) Immunofluorescence of the indicated lines of epimastigotes using anti-TcK2 (green) and anti-cruzipain (red) antibodies. The images also show the DAPI and DIC merged images. Bars = 5 μm. The figure also shows the growth curve (B) and the percentage of metacyclics after 96 h (C) of the indicated strains. (D) ROS levels were quantified by using the CellRox in non-modified parasites (+/+), in the knockout (Neo/Blas replacements, −/−) and in the add backs with the full TcK2 genes (Wild type, WT and kinase dead, KD). The quantification was also measured in the parasites maintained for 5 days in the presence of 10 μM ascorbate (Asc). The numbers in C-D are mean and standard deviation (n = 3) and asterisks indicate the relevant significant difference (p < 0.05) based on the t-Student test. (E) Western blot of the indicated lines of parasites maintained in regular medium (Control) or incubated 6 h in PBS, probed with anti-TcK2 or anti-Tubulin (Tub) antibodies. (F) Autoradiogram of a 10% SDS-PAGE containing immunoprecipitates of non-transfected epimastigotes (+/+) or the null epimastigote line (Neo/Blast, −/−) pre-incubated for 6 h at 28°C in complete LIT medium (LIT) or in PBS. The epimastigotes were lysed and the cleared extracts incubated with the rabbit anti-TcK2 and Protein G-Sepharose. The beads were washed and incubated with 2 μg/ml of 6xHis-TceIF2α in the presence of [^32^P]-γ-ATP for 1 h at 37°C. The top panel shows the autoradiogram and the bottom the gel stained with Coomassie Blue R.

To determine whether the changes observed in the null parasites were due to an indirect effect related to the absence of TcK2 as a protein, or to the requirement of an enzymatically active kinase, we generated parasites expressing the wild type and an enzymatically inactive TcK2 mutant. For this, we used null parasites obtained by replacement of the two copies of the endogenous kinase with Neo and Blast genes. This was done because these parasites were transfected with pROCK, which is a plasmid that integrates in the tubulin locus, being selected by hygromycin resistance [34]. The resulting constructs were expected to express constitutively the full length TcK2 gene. In one set of transfections the full-length wild type kinase was inserted, while in the other, a kinase with the lysine 695 replaced by alanine was used. The position of this mutation is indicated by an asterisk in S2 Fig., which corresponds to the lysine 622 of human PERK. This residue is located in the ATP binding site and inactivates the enzyme [35]. As shown in Fig. 9A, both forms of TcK2 were localized in similar vesicular structures in the posterior end of the parasite at the same position shown by cruzipain. The reinsertion of the wild type gene restored the growth defect, while the slow growth was maintained in the case of the mutated and probably inactive kinase. In addition, the differentiation capacity and ROS levels were restored by the wild type but not by the mutant kinase (Fig. 9C and D). Western blotting showed the presence of similar levels of the TcK2 protein in both cases, which were similar also to TcK2 expression levels in untransfected parasites, whereas the Neo/Blast null line that was used for these constructions has no TcK2 signal (Fig. 9E). The upper band was also seen in the kinase dead add-back. Nevertheless, upon incubation in PBS without hemin, the intensity of the upper phosphorylated band increased only in the original cells and in the wild type add-back of TcK2, not in the parasite line containing the kinase dead construct. These results indicate that the absence of the TcK2 activity is responsible for the growth defect and alterations in the oxidative metabolism observed in the null cells.

Importantly, TcK2 immunoprecipitated from epimastigotes previously incubated for 6 h in PBS had more eIF2α phosphorylation activity, as compared to the immunoprecipitates obtained from epimastigotes maintained in LIT medium (Fig. 9F). Control immunoprecipitations with null epimastigotes did not show any activity, confirming that the ability to phosphorylate the eIF2α *in vitro* was due to TcK2. Altogether, these data support the notion that the enzyme, migrating mainly as the top band is active in phosphorylating eIF2α.

## Discussion

**Figure 10:**
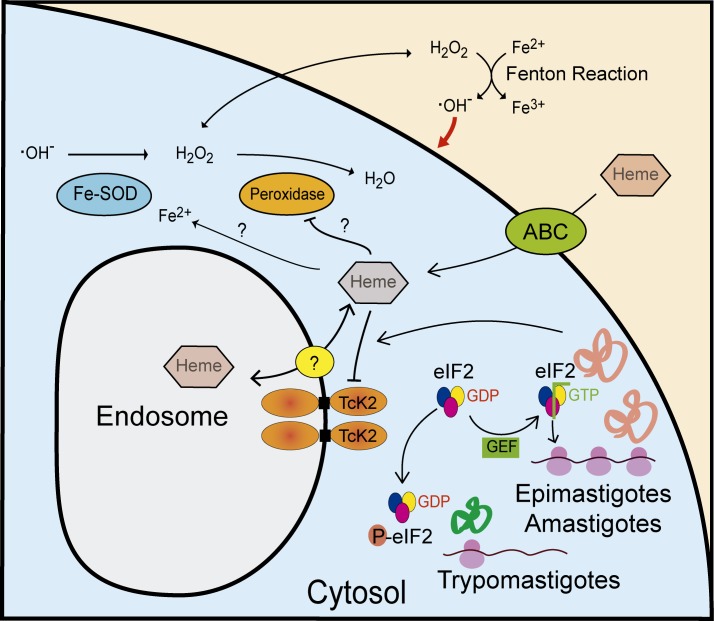
Model of the proposed actions of TcK2 in response to heme during growth and differentiation of *T. cruzi*. TcK2 is located in the membrane of endosomes and is involved in heme accumulation inside these organelles through an unknown transporter. Extracellular heme enters the cell through ABC transporters and inhibits TcK2, allowing general translation. In heme deprivation, heme is released in the cytosol, and when cytosolic levels reach low levels, TcK2 is activated leading to TceIF2α phosphorylation, decreasing general translation and allowing expression of trypomastigote specific proteins. When excess heme accumulates in the cytosol, for example in the TcK2 null parasites, FeSODs are activated and peroxidases are inhibited resulting in the production and accumulation of H_2_O_2,_ which outcomes in the increase of ROS levels by Fenton Reaction. We propose that the Fenton Reaction occurs extracellularly as ascorbate is not internalized by *T. cruzi* and washed parasites sustains better growth.

We have demonstrated that *T. cruzi* expresses a membrane bound protein kinase in the endosomes that is able to phosphorylate eIF2α in response to heme deprivation. In the absence of heme, the parasite undergoes differentiation in a process that is dependent on the active form of the kinase. In parasites lacking the kinase, heme accumulates in the cell, most likely in the cytosol, causing an increase in hydrogen peroxide levels, which leads to the generation of reactive oxygen species and parasite damage, as proposed in the model depicted in the Fig. 10. Therefore, this unusual eIF2α kinase is involved in the control of both cell differentiation and heme homeostasis in *T. cruzi*, a parasite that deals with environments presenting variable amounts of heme and other oxidant products [36].

The topology of TcK2 is very similar to PERK. Indeed, we have demonstrated that TcK2 is linked to the membrane, as shown for its orthologues in *T. brucei* [19] and *L. infantum* [21]. However, it is not predominantly in the ER, which agrees with our previous observation that *T. brucei* K2 is located in the flagellar pocket, a compartment related to the endocytic/exocytic pathway, and early endocytic vesicles [19]. In contrast to *T. brucei*, in *T. cruzi* the K2 is found in organelles more spread throughout the cytoplasm, which might reflect differences in protein trafficking in these organisms. Our immunofluorescence data strongly suggest that TcK2 is located in the endosomal membranes. Detection of the enzyme in purified reservosomes or other organelle, as well as their immunolocalization by electron microscopy could strengthen this conclusion. These findings do not exclude the possibility that some TcK2 is localized in ER mainly in the case of trypomastigotes, as there is some degree of co-localization with BiP. Further studies on endosome formation, its relationship to the ER and its remodeling during differentiation are required for a more detailed understanding of TcK2 trafficking in the cells.

Our data also suggest that TcK2 may be involved in the control of protein synthesis in the different parasite stages. We have previously shown that metacyclogenesis requires the inhibition of translation by phosphorylation of eIF2α [23]. In contrast to a cell line overexpressing a wild type form of eIF2α, a cell line overexpressing non-phosphorylatable form of eIF2α (Thr169Ala mutation) does not undergo metacyclogenesis under conditions that lack heme and other nutrients. Those previous data give strong support for the findings described here on TcK2 role in phosphorylating eIF2α *in vivo* as a requirement for differentiation.

The relatively smaller amounts of the upper band, which partially corresponds to the phosphorylated enzyme form in epimastigotes and amastigotes implies that TcK2 could be in a less active state in proliferating cells, which in fact present high rates of protein synthesis [37]. In contrast, protein synthesis is decreased in metacyclic-trypomastigotes and trypomastigotes, which is compatible with the predominant presence of relatively larger amounts of the upper phosphorylated band in the Western blotting. While epimastigotes proliferate and synthesize most of the housekeeping genes, trypomastigotes predominantly produce proteins involved in cell invasion and virulence, belonging to the trans-sialidase large family of genes and surface glycoproteins as mucins and mucin associated proteins [38]. Thus, it is possible that when hemin levels are low, the kinase is activated resulting in protein synthesis inhibition through TceIF2α phosphorylation, a requirement for *T. cruzi* differentiation, perhaps by allowing preferential translation of trypomastigote specific mRNAs, in agreement with recent findings that a subpopulation of mRNAs is translated in trypomastigotes [39].

Several other factors have been shown to promote *T. cruzi* differentiation. Examples are nutrient starvation [24], hemoglobin derived peptides [40], proteasome inhibition [41]. These factors could directly act on the differentiation, or could be part of a stress response generated by critical changes in environment, perhaps affecting heme levels or the activation of other eIF2α kinase. For example, amino acid starvation or proteasome inhibition may activate TcK1, the homolog of GCN2.

We observed that heme, but not other porphyrins, prevents parasite differentiation to metacyclic-trypomastigotes forms. In a similar way, it specifically binds and inhibits TcK2 activity *in vitro*. Specific binding was inferred by the saturable interaction as observed for HRI, which interacts more strongly to hemin (Fe^+3^-protoporphyrin) when compared to other porphyrins [42]. Further work would be necessary to precisely identify the heme binding site in TcK2. The presence of heme, but not other analogs, also promotes parasite growth by activating CaM kinase II [30]. This activation was shown to occur because of a non-deleterious increase in ROS [43].

As TcK2 is probably in the membrane of organelles, which are consumed when epimastigotes move across the insect gut, we presume that the kinase activation by heme depletion could be one key factor regulating differentiation. In fact, when parasites migrate to the midgut the amount of nutrients decrease and the electron dense structure gradually disappear [24]. As differentiation is accomplished, the content of these organelles that contain heme are consumed and cruzipain is secreted, participating in cell invasion [28, 44]. These findings are in agreement with our observations that electron lucent structures predominated in the parasites, as seen by EM in medium lacking hemin. Thus, heme levels seems to decrease initially in these organelles, then in the cytosol, most likely causing TcK2 activation on the later stages of differentiation. In fact, an increased phosphorylation of TcK2 was detected during metacyclogenesis by phosphoproteome analysis [45], supporting the notion that the enzyme is activated in trypomastigotes. Likewise, more active TcK2 was found in stressed cells compared to non-stressed epimastigotes.

The mechanism by which the organelles that accumulate heme are consumed and by which TcK2 and cruzipain disperse is unknown. It might involve events such as proteolysis, or other post-translational modifications. Interestingly, there is evidence that eIF2α phosphorylation and translation arrest are linked to membrane trafficking in mammalian cells [46], which could explain the differences in the localization and distribution of the kinase and the different endosomes.

*T. cruzi* has lost the capacity to synthesize heme, which is an essential growth factor for the parasite [47]. Heme appears to be internalized directly into the cytosol by ABC-transporters [27]. Therefore, heme accumulation in the endosomal compartment could occur through an unknown heme transporter in their membranes as a mechanism to store and prevent excessive levels in the cytosol. Alternatively, heme could be scavenged from the medium and stored in specific organelles through the parasite cytostome, as shown for other macromolecules [32]. *T. brucei* acquires heme through the haptoglobin-transferrin receptor localized in the flagellar pocket [48], where TbK2 is also localized [19]. However, the haptoglobin-transferrin receptor is found only in African Trypanosomes, which proliferate in the vertebrate blood and utilizes heme from blood. In *Leishmania amazonensis*, a parasite that develops in the insect gut and inside intracellular vacuoles, heme is taken up by the *Leishmania* heme response 1 (LHR1) protein, located in the parasite membrane and on acidic intracellular organelles [49]. A paralog of LHR1 is present in *T. cruzi* (TcCLB.511071.190, http://tritrypdb.org) and we are investigating its possible association and role in heme transport.

The generation of TcK2 null parasites confirms the kinase role in cellular differentiation, probably through the phosphorylation of eIF2α, as shown earlier [23]. We also inferred that the decrease in metacyclic-trypomastigote formation was not due to a less efficient cell growth, because the single knockout of K2 partially inhibited differentiation without affecting cellular proliferation. In addition, ascorbate that fully restored the growth rate did not fully restore differentiation. This was not the case for the cell invasion by metacyclic-trypomastigotes, which was largely affected by the absence of TcK2, and was even increased by adding ascorbate during the differentiation period. Most likely, it was due to the presence of high levels of ROS, causing parasite damage, or other modifications in the parasites that affected invasion.

Our results also showed that the lack of active TcK2 is directly related to the observed phenotypes as only the reintroduction of the wild type kinase, not of the protein mutated in the ATP binding site, abrogated the effects of its gene depletion. We also found that the incapacity to restore the normal growth phenotype and ROS was not due to protein mislocalization or poor expression. Moreover, when submitted to stress, only the endogenous and the wild type add-back TcK2 showed an increase in the upper band, which supports the notion that this band corresponds to an active and autophosphorylated kinase. Also, the presence of low levels of the upper band in the kinase dead mutant suggest that it could be phosphorylated by other kinases, in agreement with the presence of several phosphor-sites in the C-terminus of the protein [50], causing eIF2α phosphorylation. This modulation is also observed in mammalian PERK. Alternatively, it could be due to other modifications such as glycosylation, as shown in the *T. brucei* enzyme [19].

Our data also indicated that in the TcK2 null cells, the SOD dismutase activity increased and the peroxidase activity decreased, which would generate high levels of intracellular H_2_O_2_. This might occur as direct effect of the increased heme levels in the cytosol due to the failure to transport heme into endosomes. Otherwise, it could be a consequence of the differential translation mediated by increased eIF2α phosphorylation, or by phosphorylation of another substrates, as in the case of phosphorylation of the transcription factor Nrf2 in mammalian cells [51]. A possibility remains that heme is also acting as a source of iron, which can directly bind and activate superoxide dismutase, a strictly iron-dependent enzyme in trypanosomatids [52]. In addition, the reason why in the absence of TcK2 there was an increase in the heme detected in the soluble fraction after a gentle lysis is unclear at this moment. One possibility is that the kinase affects directly the heme intake into endosomes by interacting, or phosphorylating an unknown carrier. In fact, there is a link between the levels of heme and several stress elements [53], but this mechanism remains obscure. In mammalian cells, for example, PERK mediates apoptosis in response to ROS by facilitating the interaction of the ER with the mitochondria, independently of the kinase activity [54]. PERK knockouts were shown to affect largely the unfolded protein responses causing high levels of ER stress and its fragmentation [55].

The observation that ascorbate was effective at low concentrations suggests that it could protect the parasite as an antioxidant towards reactive species, donating one electron to oxidizing radicals such as hydroxyl radical [56]. This agrees with our observation that ROS levels increased in the TcK2 null parasites being restored to normal levels in cells growing in the presence of ascorbate. Therefore, it is possible that hydroxyl radicals would be generated by the Fenton reaction, which would occur with the increased levels of H_2_O_2_ produced by the parasite and minimal amounts of free Fe^2+^ present in the medium. This is in agreement with the results showing that inhibition of growth occurs a few days after ascorbate removal. At concentrations higher than 1 mM, ascorbate would act as a pro-oxidant agent, reducing Fe^3+^ to Fe^2+^, which would increase the production of more hydroxyl radicals in the presence of H_2_O_2_ by the Fenton reaction.

Heme as regulatory molecule controlling TcK2 activity also appears to occur in the mammalian stages of the parasite, as the kinase is active in trypomastigotes released from infected cells and it is mainly inactive in proliferating amastigotes. In addition, our observations indicate that intracellular amastigotes growth is largely diminished in the TcK2 null cells. The fact that growth is not restored by ascorbate could be due to its inability to decrease intracellular H_2_O_2_. These observations agree with recent findings indicating that iron and heme availability influences *T. cruzi* infection and disease progression [57]. Moreover, the intake of heme and/or iron has been found critical for *Leishmania* survival and infection [58] and several other hemoparasites [59].

In conclusion, our results indicate that heme affects the eIF2α kinase 2 of *T. cruzi* regulating development in insect and most likely in mammalian hosts. Future understanding of how this kinase regulates heme levels across the different parasite compartments and the development of specific inhibitors for this kinase could be useful in the control and in the understanding of the Chagas’ disease.

## Materials and Methods

### Ethics statement

This study was carried out in strict accordance with the recommendations in the Guide for the Care and Use of Laboratory Animals of the Brazilian National Council of Animal Experimentation (http://www.cobea.org.br/). The protocol was approved by the Committee on the Ethics of Animal Experiments of the Institutional Animal Care and Use at the Federal University of São Paulo (Id # CEP 1982/11).

### Parasite cultures and differentiation

*T. cruzi* epimastigotes (Y strain) were cultivated in liver infusion tryptose (LIT) medium supplemented with 10% fetal bovine serum at 28°C [60]. For the differentiation into metacyclic-trypomastigotes, exponentially growing epimastigotes (1 × 10^7^ cells per ml) were collected by centrifugation (2000 g for 10 min) and resuspended at a density of 5 × 10^8^ cells per ml in *Triatoma* artificial urine (TAU) medium [190 mM NaCl, 17 mM KCl, 2 mM MgCl_2_, 2 mM CaCl_2_, 8 mM phosphate buffer (pH 6.0)]. After 2 h culture at 28°C the cells were diluted to 5 × 10^6^ in TAU supplemented with 2.5% sodium bicarbonate, 500 U/ml penicillin, 10 mM L-proline, 50 mM L-glutamic acid, 2 mM L-aspartic acid, 10 mM glucose and incubated at 28°C for 96 h [37].

Intracellular amastigotes were obtained from LLCMK_2_ (ATCC CCL-7) monkey kidney cells through lysis 72 h after infection. Trypomastigotes were collected from the cell medium (from 120 to 144 h after infection) as described previously [61]. Cell invasion assays were carried out by seeding the parasites onto each well of 24-well plates containing 13-mm diameter round glass coverslips coated with 2 × 10^4^ cells. After 2 h incubation with parasites (2 × 10^5^ parasites), the medium was removed, and the wells were washed with PBS and incubated with 1 ml of 4% paraformaldehyde in PBS at room temperature for 20 min. The coverslips were then washed with PBS and mounted in Prolong Gold Antifade Reagent (Invitrogen) in the presence of 10 μg/ml of 4-6-diamidino-2-phenylindole (DAPI). Images were analyzed in an Olympus (BX-61) fluorescence microscope.

### Immunoblotting

Extracts were prepared from parasites collected by centrifugation at 2000 g for 5 min at 4°C and washed once with PBS before lysis in SDS-polyacrylamide gel electrophoresis (SDS-PAGE) sample buffer. Alternatively, extracts were prepared by lysis of 1 × 10^7^ cells in 50 μl of ice-cold 50 mM NaCl, 20 mM Tris-HCl (pH 7.4) containing 1% Triton X100, 1 mM phenylmethanesulfonyl fluoride (PMSF), and the cOmplete protease inhibitor cocktail, EDTA-free (Roche). The lysates were centrifuged at 6000 g for 10 min at 4°C and the supernatants were incubated with 5 U of calf intestinal alkaline phosphatase (Promega) for 10 min and the reaction was stopped by adding sample buffer and further incubation at 95°C for 5 min.

Immunoblots were performed with extracts of cells submitted to electrophoresis on SDS-PAGE (7.5%) and transferred to nitrocellulose membranes. The membranes were stained with 0.3% Ponceau S in 10% acetic acid and then treated with 5% nonfat milk in PBS containing 0.05% Tween-20 for 60 min. The membranes were incubated 1 h with the primary antibodies in PBS containing 0.1% Tween-20. Anti-TcK2 rabbit polyclonal antiserum, which was obtained by immunization with a recombinant protein corresponding to the kinase domain (KD) of the *T. brucei* K2 expressed in *Escherichia coli* BL21 [19]. It was used at 1:1500 dilution. A monoclonal antibody (mAb) 5D10, obtained as described in [62] by immunization with a recombinant protein corresponding to the residue 346 to 511 of *T. cruzi* K2, and expressed from pET28a (Novagen) in *E. coli* BL21. It was used at 1:500 dilution. Anti-Hsp70 [63] was used at 1:20,000, anti-*T. cruzi* β-tubulin [64] at 1:20,000 and anti-BiP [65] at 1:20,000 dilutions. The membranes were washed three times with PBS containing 0.5% Tween-20 (10 min each) followed by 1 h incubation with peroxidase labeled goat anti-mouse or rabbit IgG antibody (Invitrogen) at 1:10,000 dilutions. Bound antibodies were detected by ECL (Millipore) by using a LI-COR Odyssey imaging apparatus.

### Immunofluorescence

Parasites were washed with PBS and attached to glass slides (Tekdon Incorporated) pretreated with 0.01% poly-L-lysine for 5 min. The excess of parasites was removed and the slides incubated with 4% paraformaldehyde in PBS at room temperature for 20 min, washed in PBS and permeabilized 5 min in PBS containing 0.1% Triton X100. Fixed and permeabilized cells were washed with PBS, incubated for 20 min with PBS containing 2% BSA and for 1 h at room temperature with primary antibodies diluted in the same buffer. Anti-human influenza hemagglutinin (HA) (Covance) was used at 1:1,000, the mAb of anti-5D10 at 1:500, anti-Binding immunoglobulin protein (BiP) at 1:5,000, and anti-*T. cruzi* cruzipain mAb (a gift of Prof. Dr. Julio Scharfstein [66], at 1:2,000 dilutions. The slides were washed with PBS, incubated 1 h at room temperature with Alexa Fluor 594, or 488, goat anti-rabbit or anti-mouse IgG (Invitrogen) diluted 1:1000, washed once more, and mounted in Prolong Gold Antifade Reagent (Invitrogen) in the presence of 10 μg of DAPI per ml. Images were acquired by using a Hamamatsu Orca R2 CCD camera coupled to an Olympus (BX-61) microscope equipped with a ×100 plan Apo-oil objective (NA 1.4). Acquisitions were at every 0.2 μm for each set of excitation/emission filters. Blind deconvolution was performed by employing the AutoQuant X2.2 software (Media Cybernetics).

### Immunoprecipitation

Parasites at exponential growth phase were collected by centrifugation at 2000 g for 5 min at 4°C and resuspended at 4°C in 137 mM NaCl, 2 mM EDTA and 20 mM Tris-HCl (pH 8.0) containing protease inhibitor cocktail 1% Nonidet P40, for lysis. The lysates were centrifuged at 12000 g for 20 min and the supernatants transferred to new tubes, which were incubated at 4°C with the anti-TcK2 rabbit polyclonal antiserum for 16 h. Immune complexes were collected after 2 h incubations with Protein G Sepharose (GE). The immunoprecipitates were centrifuged at 500 g for 2 min, washed once in 150 mM NaCl, 1 mM EDTA, 1 mM EGTA, 10 mM Tris-HCl (pH 8.0) containing 1% Triton-X100.

### Electron microscopy

Parasites were fixed at room temperature with 2% formaldehyde/2.5% glutaraldehyde in 0.1 M sodium cacodylate buffer (pH 7.2). The fixed parasites were then decanted overnight on ACLAR (Electron Microscopy Sciences) film precoated with 0.01% poly-L-lysine. The ACLAR films were washed three times with 0.1 M sodium cacodylate (pH 7.2) and then fixed with 1% osmium tetroxide in the same buffer at room temperature. The preparation was then washed with cacodylate buffer followed by three washes with water. Subsequently, the ACLARfilms were treated with 0.4% uranyl acetate, gradually dehydrated in a series of ethanol solutions, and embedded in Epon resin (Electron Microscopy Sciences). After sectioning to produce consecutive sections, the material was stained with uranyl acetate and lead citrate, mounted on Formvar grids, and observed in a JEOL 1200 EX II transmission electron microscope at 80 kV.

### Generation of recombinant parasites

TcK2 knockouts were generated by gene replacement using antibiotic resistance markers flanked by untranslated regions (UTR) of the K2 gene (http://tritrypdb.org TcCLB.506559.129). The 5′-UTR region was amplified from *T. cruzi* (Y-strain) genomic DNA [67] using oligonucleotides 5k2fowApaI and 5revXbaI. The 3′-UTR was amplified using oligonucleotides 3′K2fowSal and 3K2RevSac. Primer sequences used in this study are presented in S1 Table. The amplified fragments were cloned into plasmids containing the neomycin phosphotransferase (Neo), hygromycin phosphotransferase (Hygro), or blasticidin (Blast) genes inserted into the *Xba*I and *Sal*I sites of pGEM-T (a gift from Dr. Carlos Renato Machado from the Universidade Federal de Minas Gerais, Brazil). The blasticidin gene was amplified from pNAT6Myc [68] with oligonucleotides BlastFowXbaI and BlastRevSalI. Restriction mapping and DNA sequencing confirmed all constructs.

HA-epitope tagged TcK2 was prepared in a modified pTEX plasmid [69] containing one HA encoding sequence 5-TACCCTTACGACGTTCCTGATTACGCTAGCTGA between the *Bam*H1 and *Xho*I sites. Two segments corresponding to the C-terminus sequence of TcK2 were amplified from *T. cruzi* genomic DNA (Y strain) with oligonucleotides K2TcXbaFor / K2TcNotRev and K2TcNotFor / K2TcBam and cloned sequentially into pTEX-HA to introduce a *Not* I site in the sequence and create a 900 base sequence followed by the HA tag.

Thirty micrograms of each plasmid linearized with *Not*I for the HA-tag, and *Sac*I for the gene replacements, were incubated with *T. cruzi* epimastigotes previously washed and resuspended to 5 × 10^6^ parasites in 100 μl of 137 mM NaCl, 5 mM KCl, 5.5 mM Na_2_HPO_4_, 0.77 mM glucose and 21 mM Hepes (pH 7.0) and pulsed with the Amaxa Nucleofactor (Lonza), program U-033. Cells were diluted and selected by growing in 0.2 to 0.5 mg/ml G418, 0.5 mg/ml Hygro, or 0.05 mg/ml Blast depending on the plasmid used for transfection. Limiting dilution was further used to clone the parasites.

The presence of resistance marker insertions was detected by PCR amplification of DNA extracted from parasite clones using oligonucleotides 5K2fow at the 5′UTR of K2 and G418Rev for G418, HigroR for Hygro, and BlastRev for Blast. The removal of K2 was confirmed by PCR amplification using oligonucleotides TcK2-KD-pGEX5X-1(F) and TcK2-KD-pGEX5X-1(R).

*T. cruzi* containing the original and the kinase death were obtaining by cloning both constructs in the *Xba*I and *Xho*I sites of the pROCK-GFP-HYG plasmid [34]. The full-length gene was amplified from the genomic DNA of *T. cruzi* Y strain by PCR using the primers Tck2Xba-fow and TcK2XhoI-rev. The mutated protein was generated by amplification of two fragments encompassing the full-length gene with two sets of primers. One of the fragments was generated with Pfx50 polymerase (Life Technologies) by using the primers Tck2XhoIrev and Tck2mut-fow and the other with the primers TceiF2-K2mut-rev and the TcK2Xbafow to replace the lysine 695 by an alanine. The two fragments were gel purified and assembled by a second PCR using the primers Tck2Xba-fow and TcK2XhoI-rev. The final fragment was inserted into pCR2.1 Topo (Life Technologies) and subcloned into the *Xba*I and *Xho*I sites of pROCK-GFP-HYG. The obtained plasmids were confirmed by restriction analysis and sequencing.

### Recombinant proteins, kinase and binding assays

Soluble recombinant *T. cruzi* eIF2α containing a histidine-tag was expressed from pDEST17 [23] in *E. coli* BL21. Expression was induced for 16 h at 30°C with 0.5 mM isopropyl β-D-1-thiogalactopyranoside (IPTG); the cells collected and resuspended in 0.25 M NaCl, 50 mM Tris-HCl (pH 8.0) and the cOmplete protease inhibitor cocktail and lysed by using a French press. The recombinant protein was purified by affinity chromatography on a nickel agarose column (Qiagen) by elution with the lysis solution containing 0.5 M imidazole. The kinase domain of TcK2 was amplified from total *T. cruzi* DNA with the oligonucleotides TcK2-KD-pGEX5X-1 and TcK2-KD-pGEX5X-1 and cloned into the *Eco*RI and *Xho*I sites of the plasmid pGEX5X-1 (GE) to generate a protein fused with GST. The TcK2 recombinant was expressed in *E. coli* BL21 after induction with 0.5 mM IPTG for 16 h at 30°C. The protein was purified from bacteria lysed by the French press by using affinity chromatography on GST-Sepharose (GE) equilibrated in PBS containing cOmplete cocktail, by elution in the same buffer containing 0.1 M glutathione. Both proteins were dialyzed in the kinase buffer (50 mM NaCl, 20 mM Tris-HCl (pH 7.5) at 4°C for 18 h. The kinase assays were performed in the presence of 5 μg/ml eIF2α, 1 μg/ml TcK2, 10 mM MgCl_2_, 10 μCi of [^32^P] γATP (3000 Ci/mmol, Perkin Elmer) and 0.5 mM ATP at 37°C for 1 h. When indicated the heme derivatives (Frontiers Scientific) were added 15 min before addition of ATP. Reactions were stopped by the addition of SDS-PAGE sample buffer. The labeling was quantified by using 9200 Typhon imaging apparatus (GE) after electrophoresis and Coomassie R-250 staining. For the binding assays hemin at indicated concentrations were incubated 30 min with the indicated with 5 μg/ml of recombinant proteins in 0.1 M Tris-HCl (pH 7.4) and 50 mM NaCl and the absorbance read in a SpectraMax M3 reader (Molecular Devices), at 25°C. Dissociation constant determination was estimated by using the Prisma 6 software (Graphpad).

### Reactive species of oxygen, H_2_O_2_ and peroxidases

Parasites at 2 × 10^7^ cells per ml were pre-loaded with 5 μM of CellRox (Invitrogen) in PBS for 30 min, or with 5 μM CM-H2DCFDA (Invitrogen). In the case of this later reagent, cells were further incubated for 30 min in LIT medium. Reactive species of oxygen were then detected by flow cytometry using a FACS Accuri C6 (BD Biosciences) as recommended by the manufacturer. Hydrogen peroxide levels were measured by incubating 2 × 10^7^ parasites per ml of PBS with 1 U/ml horseradish peroxidase in the presence of 2 μM Amplex Red (Invitrogen) for 30 min. The parasites were then centrifuged and the resulting fluorescence measured in the supernatants (excitation 530 nm and emission 590 nm) by using a SpectraMax M3 Reader. Total peroxidase activity was measured in the supernatant of 2 × 10^7^ parasites lysed in 50 μl of ice-cold 50 mM NaCl, 20 mM Tris-HCl (pH 7.4) containing 1% Triton X100. The activity was quantified after 30 min incubation at 25°C by adding 2 μM Amplex Red (Invitrogen) and 2 mM H_2_O_2_. SOD activity was measured in the supernatant of 1 × 10^7^ parasites previously lysed by three sonication cycles (20 sec each) in 200 μl of 50 mM potassium phosphate buffer (pH 7.4) and 0.1 mM EDTA (SOD buffer). Reactions were done at 25°C by the addition of 25 μl of the lysate into 200 μl of SOD buffer containing 10 mg/ml β-nicotinamide adenine dinucleotide and 28 mg/ml nitrotetrazolium blue chloride (both from Sigma) followed by 25 μl of 3.3 mM phenazine methosulfate (Sigma). The SOD activity was determined by the kinetics of nitrotetrazolium blue chloride reduction followed by absorption at 560 nm [70].

### Intracellular heme levels and incorporation of heme analogues

We employed the method described in [71]. Briefly, 2 × 10^7^ cells were centrifuged, washed with PBS, and resuspended in 1 ml of 10 mM Tris-HCl (pH 7.4) containing the cOmplete cocktail. The cells were lysed by freezing and thawing (three cycles), the insoluble material removed by centrifugation at 10,000 g for 10 min and 840 μl transferred to a new tube containing 100 μl of 1 M NaOH and 200 μl of pyridine. Heme levels were then measured in a 1 ml cuvette after addition of 2 mg of solid sodium dithionite at 450 nm by using the Spectra Max M3 Reader.

The incorporation of the fluorescent Zn(II) Mesoporphyrin IX (Frontier Scientific) was performed in 2 × 10^7^ parasites resuspended in 100 μl of PBS. Ten μM of the porphyrin was added and the mixture incubated for 5 min followed by the addition of the indicated concentrations of hemin as a competitor. After more 30 min, the cells were chilled by adding 800 μl of cold PBS, centrifuged and washed with cold PBS and lysed in PBS containing 0.5% Triton X-100. The fluorescent porphyrin was measured in the supernatant (excitation 405 nm and emission at 578 nm).

## Supporting Information

S1 Fig TcK2 is more soluble in trypomastigotes. Parasites were collected by centrifugation, resuspended once in PBS containing the cocktail of protease inhibitors. Increased concentrations of digitonin were added and the lysates incubated for 5 min at 37°C then with 0.3 M sucrose. Each fraction corresponds to the supernatant of 10000 g (5 min) that was mixed with SDS-PAGE sample buffer, submitted to Western Blotting and probed with the indicated antibodies.
(TIF)

S2 Fig Alignment of the kinase domains of TcK2, TcK3, PERK and HRI. The alignment was generated using the amino acid sequence from the kinases domain of TcK2 from CL-Brener sequence from residues 638–984 (TcCLB.506559.129, http://tritrypdb.org), TcK3 based on the sequence between residues 547 and 1062 of the putative kinase of *T. cruzi* Sylvio X10/1 strain (ADWP02013222, http://tritrypdb.org), the kinase domain of human PERK containing residues 599 to1073 of the NCBI gene identification 945,1 and the kinase domain of human HRI from residues 167–582 (UniProtKB/Swiss-Prot, Q9BQI3.2). The asterisk indicates the lysine 695 required for ATP binding in the catalytic site. The underlined residues are the predicted heme binding sites in TcK2 and the boxed residues the heme binding sites of HRI [3].
(TIF)

S3 Fig Heme binding is specific for TcK2. (A) Absorption spectra of heme (yellow line), GST + hemin (blue line) and GST-TcK2 (green line), GST-TcK2 incubated with hemin (black line) and GST-PfK4 (red line). (B) SDS-PAGE of His6x-TceIF2α incubated without (−) or with (+) 10 μM hemin in the presence of GST-TcK2, or GST-PK4 for 30 min with [^32^P]-γ-ATP. The upper gel shows the autoradiogram and at the bottom is the same gel stained with Coomassie Blue R250.
(TIF)

S1 Table List of synthetic oligonucleotides used in this work and their respective sequence. (DOCX)
