# Supplementary figures and images for: A Membrane-bound eIF2 Alpha Kinase Located in Endosomes Is Regulated by Heme and Controls Differentiation and ROS Levels in Trypanosoma cruzi
Source: PLoS Pathog. 2015 Feb 6;11(2):e1004618. doi: 10.1371/journal.ppat.1004618 (PMC4450062; doi:10.1371/journal.ppat.1004618)

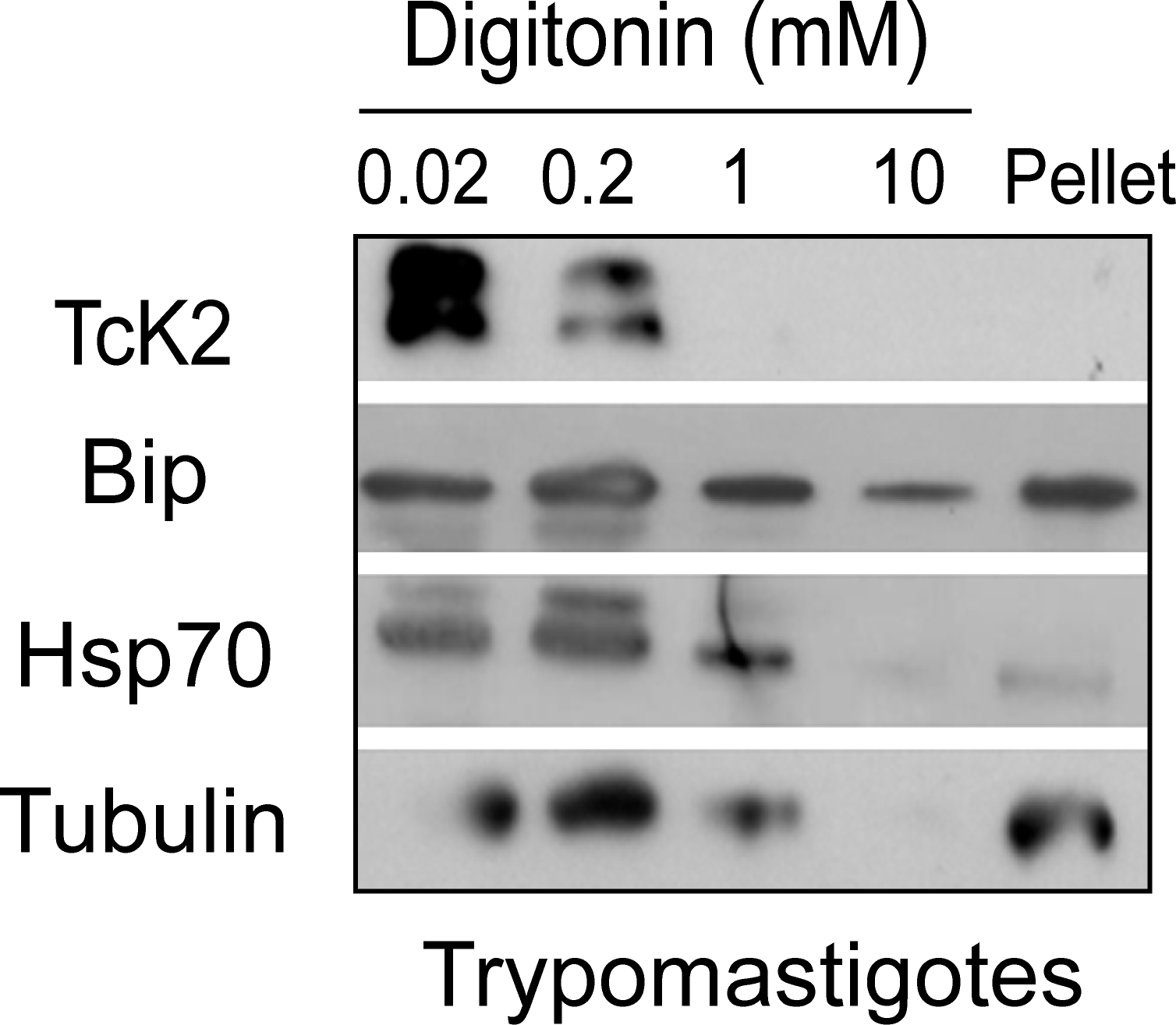

Supplement: S1 Fig — Parasites were collected by centrifugation, resuspended once in PBS containing the cocktail of protease inhibitors. Increased concentrations of digitonin were added and the lysates incubated for 5 min at 37°C then with 0.3 M sucrose. Each fraction corresponds to the supernatant of 10000 g (5 min) that was mixed with SDS-PAGE sample buffer, submitted to Western Blotting and probed with the indicated antibodies. (TIF) [file ppat.1004618.s001.tif]

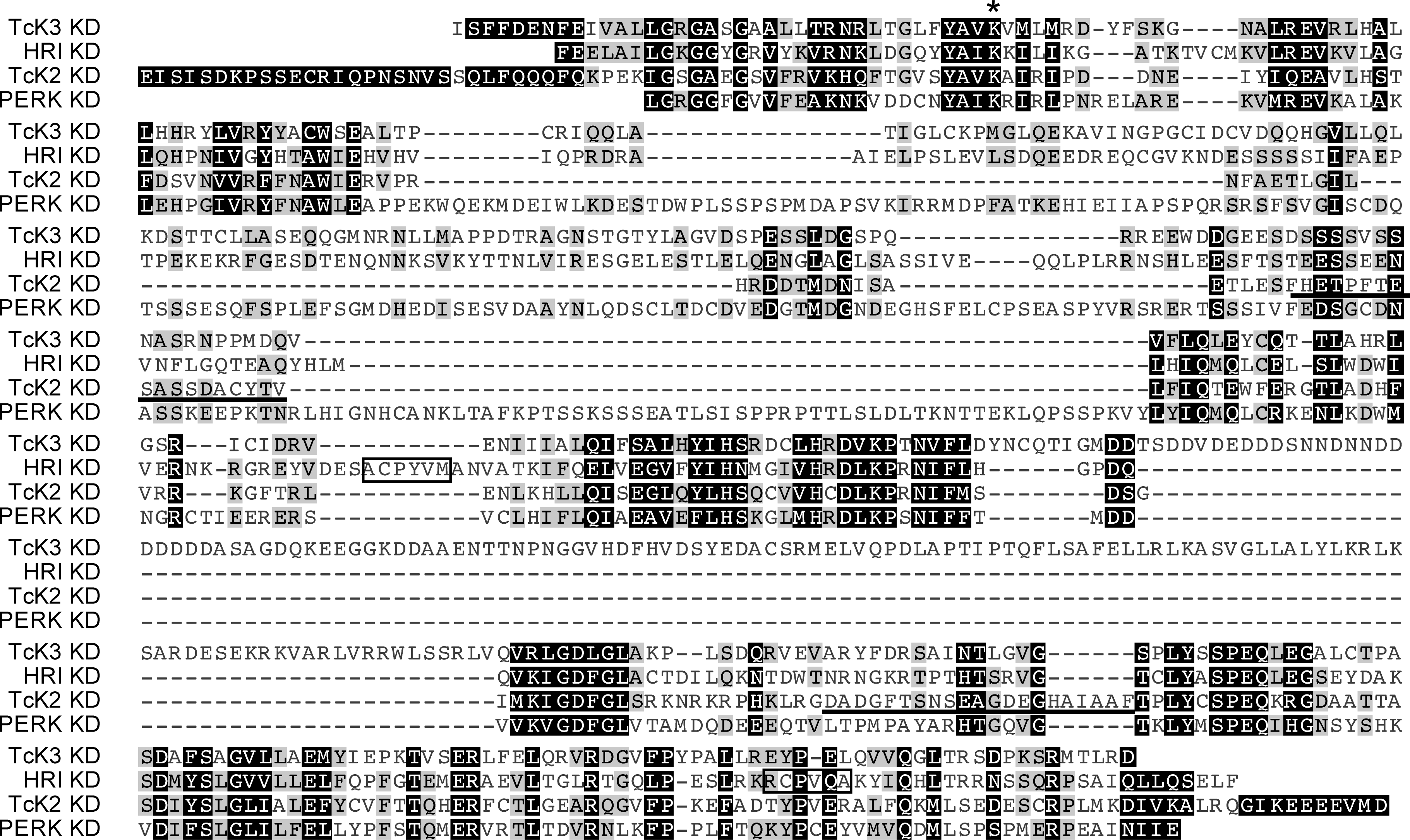

Supplement: S2 Fig — The alignment was generated using the amino acid sequence from the kinases domain of TcK2 from CL-Brener sequence from residues 638–984 (TcCLB.506559.129, http://tritrypdb.org), TcK3 based on the sequence between residues 547 and 1062 of the putative kinase of T. cruzi Sylvio X10/1 strain (ADWP02013222, http://tritrypdb.org), the kinase domain of human PERK containing residues 599 to1073 of the NCBI gene identification 945,1 and the kinase domain of human HRI from residues 167–582 (UniProtKB/Swiss-Prot, Q9BQI3.2). The asterisk indicates the lysine 695 required for ATP binding in the catalytic site. The underlined residues are the predicted heme binding sites in TcK2 and the boxed residues the heme binding sites of HRI [3]. (TIF) [file ppat.1004618.s002.tif]

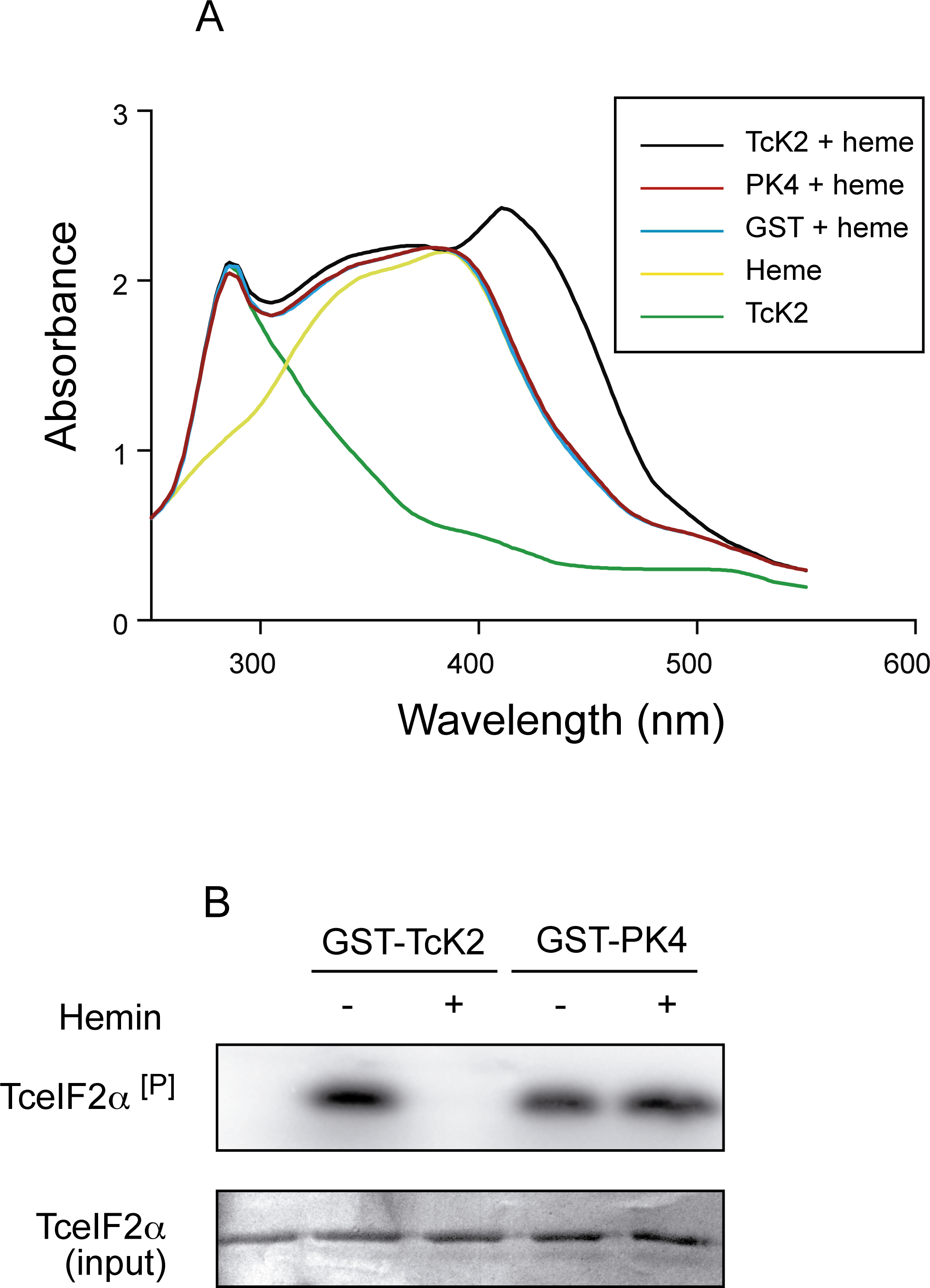

Supplement: S3 Fig — (A) Absorption spectra of heme (yellow line), GST + hemin (blue line) and GST-TcK2 (green line), GST-TcK2 incubated with hemin (black line) and GST-PfK4 (red line). (B) SDS-PAGE of His6x-TceIF2α incubated without (−) or with (+) 10 μM hemin in the presence of GST-TcK2, or GST-PK4 for 30 min with [32P]-γ-ATP. The upper gel shows the autoradiogram and at the bottom is the same gel stained with Coomassie Blue R250. (TIF) [file ppat.1004618.s003.tif]
