# Supplementary material for: A Membrane-bound eIF2 Alpha Kinase Located in Endosomes Is Regulated by Heme and Controls Differentiation and ROS Levels in Trypanosoma cruzi
Source: PLoS Pathog. 2015 Feb 6;11(2):e1004618. doi: 10.1371/journal.ppat.1004618 (PMC4450062; doi:10.1371/journal.ppat.1004618)
Supplement: S1 Table — (DOCX) [file ppat.1004618.s004.docx]

| Oligonucleotide | Sequence |
| --- | --- |
| 5k2fowApaI | 5’-GGGCCCACACGGAAGCGAGCAAACAC |
| 5revXbaI | 5’-AGATCTTTAGGGAAAAATGCCACAG |
| 3’K2fowSal | 5’-GTCGACTCAAAACACATGACGCGC |
| 3K2RevSac | 5’-GAGCTCTTCCATTGGTGTCCATC |
| BlastFowXbaI | 5’-TCTAGAATGGCCAAGCCTTTGTCTC |
| BlastRevSalI | 5’-CAGGTCGCTATGTTCAGTCCAC |
| K2TcXbaFo | 5’-TCTAGAATCTGCCTCCAGTGATGG |
| K2TcNotRev | 5’-GGCCGCTTACGATTTTTTCTC |
| K2TcNotFor | 5’-GCGGCCGCATAAATTGCGCG |
| K2TcBam | 5’-GGATCCTGTGTTTTGAGTAAACTC |
| 5K2fow | 5’- GGGCCCACACGGAAGCGAGCAAACAC |
| G418Rev | 5’-AAGCTTGAAGAACTCGTCAAGAAGGCGATA |
| HigroR | 5’-CAGCTGGATAAGGAAACGGG |
| BlastRev | 5’-CAGGTCGC TATGTTCAGTCCAC |
| TcK2-KD-pGEX5X-1(F) | 5’- GAATTCCAGCAGTTCCAAAAGCCCGA |
| TcK2-KD-pGEX5X-1(R) | 5’- CTCGAGTCGACCCGGCTGTCGCAGGGCTTTAACGA |
| Tck2Xba-fow | 5’ GCTCTAGATGCCGGATGCCTCTATTCG |
| TcK2XhoI-rev | 5’-GCCTCGAGTCATGTGTTTTGAGGTAAAC |
| Tck2mut-fow | 5’-CCTACGCCGTCGCGGCTATTCGTATACCAGATG |
| TceiF2-K2mut-rev | 5’-CATCTGCTATACGAATAGCCGCGACGGCGTAGG |

Supplementary Table I
